# High-throughput functional screen identifies YWHAZ as a key regulator of pancreatic cancer metastasis

**DOI:** 10.1038/s41419-023-05951-5

**Published:** 2023-07-14

**Authors:** Fang Cao, Yunpeng Jiang, Lin Chang, Hongzhen Du, De Chang, Chunxiao Pan, Xiaozheng Huang, Donglin Yu, Mi Zhang, Yongna Fan, Xiaocui Bian, Kailong Li

**Affiliations:** 1 Key Laboratory of Carcinogenesis and Translational Research (Ministry of Education), Department of Pathology, Peking University Cancer Hospital & Institute, Beijing, China; 2 Department of Biochemistry and Biophysics, Beijing Key Laboratory of Protein Posttranslational Modifications and Cell Function, School of Basic Medical Sciences, Peking University Health Science Center, Beijing, China; 3 Key Laboratory of Carcinogenesis and Translational Research (Ministry of Education), Department of Endoscopy Center, Peking University Cancer Hospital & Institute, Beijing, China; 4 Department of Pathology, Cell Resource Center, Institute of Basic Medical Sciences, Chinese Academy of Medical Sciences (CAMS) & School of Basic Medicine, Peking Union Medical College (PUMC), Beijing, China; 5 Department of Pulmonary and Critical Care Medicine, 7th Medical Center of Chinese PLA General Hospital, Beijing, China

**Keywords:** Metastasis, Transposition

## Abstract

Pancreatic cancer is a leading cause of cancer death due to its early metastasis and limited response to the current therapies. Metastasis is a complicated multistep process, which is determined by complex genetic alterations. Despite the identification of many metastasis-related genes, distinguishing the drivers from numerous passengers and establishing the causality in cancer pathophysiology remains challenging. Here, we established a high-throughput and *piggyBac* transposon-based genetic screening platform, which enables either reduced or increased expression of chromosomal genes near the incorporation site of the gene search vector cassette that contains a doxycycline-regulated promoter. Using this strategy, we identified YWHAZ as a key regulator of pancreatic cancer metastasis. We demonstrated that functional activation of *Ywhaz* by the gene search vector led to enhanced metastatic capability in mouse pancreatic cancer cells. The metastasis-promoting role of YWHAZ was further validated in human pancreatic cancer cells. Overexpression of YWHAZ resulted in more aggressive metastatic phenotypes in vitro and a shorter survival rate in vivo by modulating epithelial-to-mesenchymal transition. Hence, our study established a high-throughput screening method to investigate the functional relevance of novel genes and validated YWHAZ as a key regulator of pancreatic cancer metastasis.

## Introduction

Pancreatic ductal adenocarcinoma (PDAC) is the most common type of pancreatic cancer [1]. It is the fourth leading cause of cancer death in the United States [2]. Its high mortality rate might be due to the late diagnosis of most PDAC patients with locally advanced tumors and/or metastases [3, 4]. Therefore, the molecular pathogenesis of pancreatic cancer metastasis is needed to be investigated for developing novel effective therapeutic strategies and clinical management.

Previous studies have demonstrated that metastasis is a multistep process, which is controlled by a series of genetic and epigenetic events in both the tumor cells and tumor microenvironment, as indicated in the “seed and soil” hypothesis [5–9]. Numerous histological and molecular studies conducted on the progression of PDAC have suggested that PDAC evolves from a pre-invasive state known as pancreatic intraepithelial neoplasia (PanIN) to the advanced aggressive PDAC, which is accompanied by frequent mutations in *KRAS* and certain tumor suppressor genes [10–15]. The mutations in the *KRAS* gene are the first-noticed genetic alterations in pancreatic cancer with a more than 80% mutation frequency in the advanced PDAC along with the inactive mutations in *CDKN2/INK4A* (90–95%), *TP53* (50–85%), and *DPC4/SMAD4* (50%) [16–19]. Consistent with these findings, the transgenic mice engineered with pancreas-specific *Kras*^*G12D*^ mutation developed classic PanIN lesions, which rapidly progressed to a highly invasive and metastatic PDAC when another oncogene *Src* was activated or certain tumor-suppressing genes, such as *Ink4a*, *Tp53* or *Smad4*, were inactivated [20–24]. The increasing studies, showing these genes as drivers for PDAC development and metastasis, have shown the need of developing therapeutic drugs to target these genes for cancer therapy [25–27]. However, to date, effective anticancer drugs with promising outcomes in clinics are still lacking. In addition, recent high-throughput sequencing analyses have reported thousands of low-frequency somatic genetic mutations in clinical samples [10, 28, 29]. Most of these mutations have uncertain functional relevance to PDAC metastasis [30], suggesting that the identified signature mutations might represent a small part of the genes which drive the development and metastasis of PDAC. The identification of novel “driver” genes or pathways from this complicated genetic background remains a big challenge in cancer research.

Genome-wide screening methods (RNAi, ORF, CRISPR, etc.) are efficient ways to explore key genes involved in tumor progression. Applying a direct in vivo RNA interference (RNAi) strategy to screen for genes that upon repression predispose mice to squamous cell carcinomas (SCCs), Schramek and his colleagues unveiled myosin IIa as a tumor suppressor of SCCs [31]. To identify genes that can functionally substitute for oncogenic *RAS*, Shao and his colleagues systematically expressed more than 15 thousand open reading frames in a human KRAS-dependent colon cancer cell line and found that KRAS and YAP1 converge to regulate EMT and tumor survival [32]. In recent years, CRISPR (clustered regularly interspaced short palindromic repeats) was used to interrogate the gene function on a genome-wide scale. Based on a CRISPR interference platform, it was found that NF1, MED12, NF2, CUL3, TADA2B and TADA1 were involved in resistance to vemurafenib in melanoma [33], and functional loss of *Nf2*, *Pten*, *Trim72* and *Cdkn2a* induced tumor growth and metastasis [34]. Furthermore, the CRISPR activation screen was used to identify BCL-2 proteins and B3GNT2 as drivers of cancer resistance to T cell-mediated cytotoxicity [35].

The phenotype-driven genomic screen, which is developed using transposon-tagged mutagenesis, has been shown to be an effective strategy to identify the potential target genes in disease pathogenesis [36]. Unlike the loss-of-function screening based on RNA interference or gain-of-function screening based on the cDNA libraries, the transposon can achieve mutagenic effects both in the coding and non-coding regions [37, 38]. Using the *Sleeping Beauty* (SB) transposon-mediated insertional mutagenesis, Pérez-Mancera and colleagues revealed novel candidate genes, including the X-linked deubiquitinase *Usp9x*, a newly defined tumor suppressor, which could accelerate the tumorigenesis and progression of PDAC in a transgenic mouse model with PanIN lesions in cooperation with the oncogenic *Kras*^*G12D*^ [39]. Another transposon *piggyBac*, which was originally isolated from the cabbage looper moth Trichoplusiani [40], is a promising alternative for creating insertional mutations. As compared to the SB, *piggyBac* showed greater integration efficiency, lesser inhibition of overproduction, and a higher integration preference in the regions near transcriptional start sites (TSS) and also within the long terminal repeats [41]. *PiggyBac* was recently engineered and became highly active in many cell types and could also mediate the long-term expression in mammalian cells in vivo [36, 42–49]. The *piggyBac-based* functional genetic screens have identified many cancer-related genes involved in tumorigenesis, which could not be identified in the SB transposon-based screens [50]. Therefore, *piggyBac*-mediated mutagenesis is a promising approach for developing functional genomic screens to identify the potential metastasis-controlling targets in PDAC.

In this study, we developed a random gene perturbation method using a piggyBac transposon system to screen the causal genes in pancreatic cancer metastasis. Specifically, a mutagenesis library, containing more than 500,000 genomic integration events, was initially established. The highly metastatic lesions were then screened in the mouse models, and the causality of metastatic phenotypes with the genetic mutations was validated by turning on/off the mutagenic effects using a Tet-off system in the gene search vector. We finally identified a total of 36 targeted genes and 4 non-coding RNAs as the candidate regulators of metastasis, including *Errfi1, Vmp1, Anxa2, Ywhaz* and *Anxa3*. The functional role of YWHAZ was further validated both in mouse and human cell lines. We found that overexpression of YWHAZ resulted in more aggressive metastatic phenotypes in vitro and a shorter survival rate in vivo through a mechanism of EMT. Thus, we established a novel high-throughput screening strategy to uncover the genetic determinants of metastasis. These genes might provide deeper insights into PDAC metastasis, thereby providing novel targets for the development of cancer therapy.

## Materials and methods

### Cell lines and plasmids

The mouse pancreatic cancer cell line Pan02 and human pancreatic cancer cell lines AsPC-1 and Panc1 were obtained from the Cell Resource Center, Peking Union Medical College (which is the headquarter of National Science & Technology Infrastructure-National BioMedical Cell-Line Resource, NSTI-BMCR). Cells were cultured in DMEM (HyClone) supplemented with 5% fetal bovine serum (FBS, HyClone), 100 units/ml penicillin, 100 μg/ml streptomycin and cultured at 37° C in a humidified atmosphere with 5% CO_2_. *PiggyBac* transposon-mediated gene search vector (PB-GSV) and pCAG-tTA plasmid were constructed as described previously [51]. *PiggyBac* transposase vector (mPB) was a gift from Dr. Alan Bradley (Sanger Center, UK), the tetracycline-inducible pTRE-luciferase plasmid and lentiviral vector pLV-TRE-GFP were constructed as described previously [52].

### Construction of the Tet-off system and luciferase activity assay

Approximately 1 × 10^5^ Pan02 cells were seeded into each well of a 24-well plate for 24 h before transfection. The linear pCAG-tTA plasmid (0.5 μg) was transfected into each well using Fugene ^®^ HD (Promega, USA) transfection reagent following the manufacturer’s instruction. 24 h after transfection, the cells were trypsinized and passaged onto a 10 cm plate with medium containing 2 μg/ml puromycin (InvivoGen, USA). After one week, the puromycin-resistant clones were isolated. Each clone was transfected with pTRE-luciferase plasmid and carrier DNA pSP72 (Addgene plasmid). After 8 h, the cells were treated with (+) or without (−) 2 μg/ml doxycycline (Dox, Sigma, USA) for 48 h. Then, the cells were lysed using harvest buffer (1 M Tris–HCl pH 7.5, 1 M DTT, and 10% Triton X-100) and mixed with luciferase substrate buffer of equal volume [53] (luciferin, GOLD Biotechnology). Light emissions were measured using a Luminometer (TURNER Modulus, American) following the manufacturer’s instructions, and the relative luciferase activities were calculated by subtracting the light measurement of cells without transfection.

### Generation of mutagenic libraries

Pan02-4B3 cells were cultured and seeded into a 24-well plate. 25 ng PB-GSV, 2.5 ng mPB, and carrier DNA pSP72 were transfected into the cells. The positive PB-GSV transfectants were then selected with G418 (Calbiochem, Germany). After 2 weeks, the visible colonies were counted. For the construction of a mutagenic library on a large scale, a total of 30,000–60,000 colonies were trypsinized, mixed, and frozen as small libraries. A total of 2–3 libraries were further mixed and injected into the mice using four different methods for in vivo screening.

To explore the genomic coverage of the libraries, we isolated 102 clones from two libraries (L1 and L2) and identified the integration events by Splinkerette PCR followed by sequence analysis. The integration efficiency (the number of integration events in different genes relative to the total clones) was used to estimate the number of clones required to saturate the genome. To include more genes in the screening pool, we finally constructed 7 libraries that contained more than 530,000 G418-resistant clones with PB-GSV integrations, theoretically 10-fold needed. For mice used for screening, we aimed to ensure that each mutant clone had more than 100 cell copies injected into animals by any of the injection methods, which were summarized in Table S2.

### Establishment of the PDAC mouse models, tumor formation and metastasis analysis

For in vivo screening of metastasis, various PDAC mouse models were established. Six-week-old female C57BL/6 mice, purchased from Beijing Huafukang Biotechnology Co., Ltd., were housed in cages (*n* = 6 per cage) in a specific-pathogen-free environment. The mycoplasma-free cells were carefully harvested and dissociated to single-cell suspensions. Approximately 5 × 10^5^–2.5 × 10^6^ cells were suspended in PBS and injected into both dorsal flanks of the mice for subcutaneous transplantation (s.c. models) or injected into the abdominal cavity at the middle-lower quadrant of the mice for intraperitoneal transplantation (i.p. models). Similarly, approximately 2 × 10^5^–1 × 10^6^ cells were suspended in PBS and injected into the lateral tail vein of the mice for intravenous transplantation (i.v. models). For establishing the orthotopic PDAC model, the mice were first anesthetized. An incision was made in the middle-left abdomen and the spleen was exteriorized with the tail of the pancreas (o.r. models). A total of 3 × 10^5^–1 × 10^6^ cells, suspended in 50 μL D-Hanks buffer with 1% (vol/vol) Matrigel basement membrane matrix (BD Biosciences, USA) and maintained on ice, were then injected into the tail of the pancreas [54, 55].

To evaluate the tumor growth in vivo, the volume of the subcutaneous tumor was measured every 7 days using a Vernier caliper. For the metastasis study, all the animals were sacrificed and examined when cachexia or dyspnea developed. While for the survival study, the individual mouse was recorded after death. The visible organ metastatic nodules were either dissected for primary culture or fixed with a 10% neutral-buffered formalin solution for the histological analysis. Macro-metastasis was defined as visible lesions grossly and/or the diameter of the tumors measured under the microscope equal to or more than 1 mm. The lesions less than 1 mm in diameter were called micro-metastasis [31]. Tumor thrombi in vessels were ruled out for micro-metastasis. All the animal experiments were conducted according to the guidelines for the care and use of laboratory animals and approved by the Animal Ethics Committee at the Institute of Basic Medical Sciences, Chinese Academy of Medical Sciences (CAMS) & Peking Union Medical College (PUMC), and Peking University Health Science Center.

### Primary culture

Big visible metastatic lesions from each organ were separately scissored out for primary culture immediately after the mice were sacrificed. If there were diffuse small nodules impossible to isolate or no visible metastasis in organs like lungs, livers, and brains, the entire organs were dissected and subjected to primary culture. The tissues were rinsed with cold PBS (4 °C) and cut into fine sections using two scalpels. These tissue sections were then digested with collagenase type IV (200 U/ml, Invitrogen, USA) in DMEM medium without FBS at 37 °C for 3–5 h. After the enzymatic digestion, the cells were washed and then cultured in DMEM with 5% FBS, 200 units/ml penicillin, and 0.2 mg/ml streptomycin for 48h. Positive PB-GSV-transfected cells were selected by adding 800 μg/ml G418 to the culture medium for 2–3 weeks. One lesion or organ in the culture dish was defined as a highly metastatic subpopulation in the screening process and a metastatic subclone in the following validation process.

### Splinkerette and genomic PCR

Genomic DNA was extracted from the highly metastatic subclones. Splinkerette adaptors, primers for the adaptors [56], and both terminal repeats of the *piggyBac* transposon (two for each 3’PB-TR and 5’PB-TR) [51] were designed (summarized in Supplementary Table S1). Splinkerette PCR and sequence alignment were performed as described previously [51]. The PB-GSV-targeted genes were further validated using genomic PCR and Sanger sequencing. All the primers are listed in Supplementary Table S1.

### Pathology analysis and immunohistochemistry or immunofluorescence analysis

The samples from primary tumors and metastatic organs were fixed in a 10% neutral-buffered formalin solution, embedded in paraffin, and sliced into 3 μm-thick sections for HE staining or immunochemistry analysis. For the immunochemistry analysis, the deparaffinized and rehydrated tumor tissue sections were heated in the boiled sodium citrate buffer (0.01 M, pH 6.0) for 5 min to retrieve the antigens and treated with 3% hydrogen peroxide in PBS to block the endogenous peroxidase. The tumor tissue sections were incubated with the primary antibodies without washing for 12 h at 4 °C after blocking. YWHAZ (1:2500, ab51129, Abcam, Cambridge, UK), E-Cadherin (24E10, 1:100), N-Cadherin (D4R1H, 1:100), Claudin-1 (D5H1D, 1:100), β-Catenin (D10A8, 1;100), ZO-1 (D7D12, 1:100), Snail (C15D3, 1:100), Slug (C19G7, 1:100), ZEB1 (D80D3, 1:100), and Ki-67 (D3B5, 1:400) were purchased from Cell Signaling Technology, USA.

Tumor tissue sections were incubated with the peroxidase-conjugated secondary antibodies (anti-rabbit IgG, 1:200, ZSGB-BIO, China) at room temperature for 1 h. Diaminobenzidine reactions (ZSGB-BIO, China) and Mayer’s hematoxylin staining (ZSGB-BIO, China) were performed using the standard procedure. The LEICA DM3000 LED system (UK) was used for microscopic observation. The characteristic images were obtained using the P250 FLASH Scanning System along with the CaseViewer software (3D HISTECH, Ltd, China). YWHAZ (1:400) was further detected in the cell climbing sheets and the second antibody of goat anti-rabbit IgG H&L (Alexa Fluor 647, ab150083, Abcam, Cambridge, UK) was used for the immunofluorescence analysis. Confocal microscopy (TCS SP2AOBS, Leica, Germany) was used for observation and image capturing. The immunochemical results were recorded including the location (membrane, cytoplasmic or nuclear staining), the percentage (0–100%), and the intensity (weak, moderate, and strong positivity) in a semi-quantification analysis. An immunoreactive score (IRS) was calculated as previously described [57]. The expression levels were classified into low expression (0–8 points) and high expression (9–12 points). The positive cells and metastatic lesions were confirmed by at least two pathologists.

### Lentiviral transduction

For the overexpression of YWHAZ, the human *YWHAZ* and mouse *Ywhaz* cDNAs (NCBI, human Gene ID: 7534, and mouse Gene ID: 22631) from either the AsPC-1 or Pan02 cells were amplified. The amplified DNA fragments were digested using *Bam*H1 and *Xba*1 and then cloned into the pLV-EF1α-IRES-EGFP vector. All the lentiviral vectors were constructed and packaged. The target cells including Pan02, AsPC-1, and Panc1 cells were infected. The GFP-positive cells were sorted by fluorescent sorting (BD FACS Aria, USA). All the primer sequences are listed in Supplementary Table S1.

### Western blotting

Cells were harvested with the lysis buffer, containing 7 M urea, 100 mM NaH_2_PO_4_, 10 mM Tris–HCl, 1 mM PSMF, and proteinase inhibitor. The protein solutions were prepared on ice, separated using SDS–PAGE, and transferred to NC membranes (0.45 μm, Millipore). The immunoblotting was performed using the diluted primary and secondary antibodies (YWHAZ, 1:3500, Santa Cruz; E-cadherin, 1:2000, CST; β-Tubulin, 1:3000, CST; Snail, 1:1000, CST; Phospho-Akt (Ser473), 1:1000, CST; anti-rabbit IgG, 1:3000, ZSGB-BIO; Vimentin (D21H3), 1:1000, CST; N-Cadherin, 1:1000, CST; ZO-1,1:1000, CST; ZEB1, 1:1000, CST). β-Tubulin or β-actin was used as a loading control. The results were detected using an Enhanced Chemiluminescence (ECL) system.

### RNA preparation and quantitative real-time PCR

Total RNA was extracted using TRIzol reagent (Invitrogen, USA), and cDNA was synthesized using the iScript cDNA Synthesis Kit (BIO-RAD). An equivalent volume of cDNA was used to perform quantitative real-time PCR (qPCR) using SsoFast EvaGreen Supermix (BIO-RAD) following the manufacturer’s instructions. The specific primer sequences for each target gene are listed in Supplementary Table S1 [58]. The fold change was calculated by normalizing to *GAPDH*.

### RNA-seq analysis

RNA-seq library was prepared using NEBNext® Ultra™ RNA Library Prep Kit for Illumina. Sequencing reads from all RNA-seq experiments was aligned to the mouse reference genome (GENCODE Version M9) by STAR 2.5.2b with the parameters: –outFilterMultimapNmax 1. Differentially expressed genes were identified by DESeq2. The differentially expressed genes at each stage were analyzed by GO enrichment using ClusterProfiler.

### Transwell invasion assay

For the transwell invasion assay, Matrigel was coated on each transwell filter (8 μm pore size, Costar) before cell seeding. Cells were seeded on the top of Matrigel at a density of 2 × 10^5^ cells per well. The filters were swabbed to clean the cells and Matrigel upside, fixed in 4% paraformaldehyde, and then stained with crystal violet solution. Invaded cells were counted in at least 10 areas under the microscope.

### Soft agar assay

DMEM with 10% FBS and 0.6% agarose (Invitrogen, USA) was added into each well of a six-well plate for 4 h before cell seeding. Then, single-cell suspension, containing 2 × 10^3^ cells, 10% FBS, and 0.5% agarose in DMEM, were carefully seeded into each well. The culture media, containing 10% FBS, was added to the top layer after 8 h and changed every 3 days. The visible cellular colonies on the top layer were calculated after culturing for 30–40 days.

### Statistical analysis and HPA data analysis

All the data were statistically analyzed using SPSS 20.0 for Windows (SPSS, Inc., Chicago, IL) and presented as mean ± SEM unless indicated otherwise. The correlations of gene expression with the survival rate of animals were analyzed using Kaplan–Meier analysis and other data analyses were performed using the Student’s *t*-test. The statistical significance was defined as *p* < 0.05. 173 cases of clinical pancreatic cancer samples with variables such as age, gender, gene expression, stage, and overall survival time, were extracted from the human protein atlas (HPA) database or the cancer genome atlas program (TCGA) database. Univariate survival analysis was conducted using Kaplan–Meier survival analysis. Multivariate analysis was conducted using COX regression analysis.

## Results

### Establishment of *piggyBac* transposon-mediated high-throughput mutagenic libraries

**Fig. 1:**
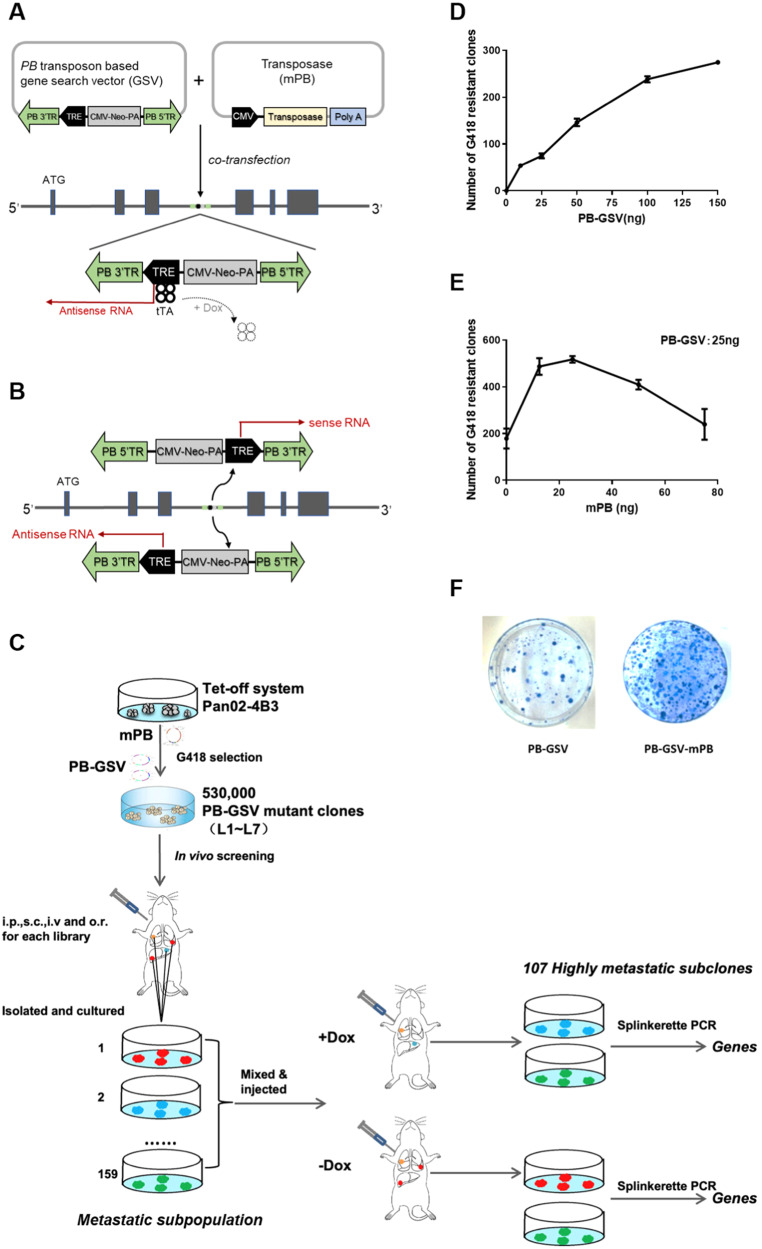
Establishing the regulated high-throughput mutagenic libraries. **A** *piggyBac* transposon-based gene search vector (PB-GSV) contains TRE (tetracycline response element) and CMV-Neo-PA (neo-expression cassette). PB-GSV could be efficiently integrated into the genome when co-transfected with transposase mPB. Tetracycline transactivator (tTA) could initiate mutagenesis when binding to the TRE element. This mutagenic effect was turned off in the presence of tetracycline (+Dox) (Tet-off system). The *piggyBac*-based mutagenic units combined with the Tet-off system resulted in a regulable high-throughput mutagenic system. **B** Different mutagenic effects were initiated by the sense RNA or antisense RNA depending on the integration orientation of PB-GSV in the genome. **C** Flow chart illustrates the screening including the establishment of mutagenic libraries, screening metastatic lesions in mouse models, functional validation of the metastatic ability of the subpopulations cultured from the metastatic lesions in the first-round screening in *vivo* by turning off the mutagenesis through the Tet-off system. The metastatic lesions in the second-round screening in vivo were cultured, and the integration sites were analyzed. **D** Titration of the transfection efficiency of the gene search vector. **E** High integration efficacy was achieved with an optimized ratio of PB-GSV and mPB. **F** The integration efficacy was represented by the number of G418-resistant clones stained by methylene blue.

To develop a functional genetic screen method, we combined *piggyBac* transposon mutagenesis, Tet-inducible expression system, and antisense RNA technology [51, 59]. Specifically, a gene search vector was constructed based on *piggyBac* transposon (PB-GSV), which contained a tetracycline-regulated element (TRE) regulated promoter. PB-GSV could be efficiently integrated into the genome specifically at ‘TTAA’ sites randomly dispersed in the genome with the help of mPB vector which expresses transposase. In the absence of tetracycline, the tetracycline transactivator (tTA) binds to TRE and initiates the sense or antisense transcription, then enhance or blocks the target gene expression. The addition of Dox will reverse the production of either sense or antisense RNA by detaching tTA from TRE, thus reversing the target gene expression and phenotypes. Hence, the *piggyBac*-based mutagenic units combined with the Tet-off system resulted in a regulable high-throughput mutagenic strategy (Fig. 1A). The gain-of-function or loss-of-function mutagenesis, initiated by the sense or antisense RNA, was determined by the integration orientation of PB-GSV in the genome [60] (Fig. 1B). The in vivo screening was then performed to identify and validate the causal genes in metastasis, the workflow of the experiment, including the establishment of the library, in vivo screening, primary culturing of dissected metastatic lesions, and in vivo validation by regulated mutagenesis, are shown in the flow chart (Fig. 1C).

To identify the key regulators involved in pancreatic cancer metastasis, a regulated two-element mutagenic system was established in Pan02 cells, which was developed by implanting cotton thread-carrying 3-methylcholanthrene into the pancreas of the C57BL/6 mouse. The orthotopic transplantation of Pan02 cells shows low organ metastasis [54]. Pan02 cells were firstly transfected with pCAG-tTA plasmid and a total of 306 puromycin-resistant clones were selected for the analysis of luciferase activity to evaluate the induction efficiency. In a subclone Pan02-4B3, the luciferase activity was reduced by more than 250-fold with Dox treatment (Fig. S1A). When transfected with pLV-TRE-EGFP, Pan02-4B3 showed higher expression of GFP, which sharply decreased after treatment with Dox (Fig. S1B, C). To investigate the regulation of GFP expression by Dox treatment in vivo, the pLV-TRE-EGFP-transfected Pan02-4B3 cells (Pan02-4B3-GFP) were transplanted into the tail of the mouse pancreas, which was an orthotopic mouse model and the injection method was summarized in the “Materials and methods” section. The immunohistochemical analysis of tumor tissues indicated that the GFP expression decreased significantly after 40 days by supplementing Dox in drinking water (2 mg/ml in 1% sucrose) (Fig. S1D). These findings demonstrated a successful establishment of a well-regulated Tet-off system in the Pan02 cell line both in vitro and in vivo.

The amount of PB-GSV was then explored to achieve minimal random integration (Fig. 1D). The highest insertion efficacy was achieved with the optimal ratio of PB-GSV and mPB, namely 25 ng PB-GSV and 12.5 ng mPB for 6 × 10^4^ cells, determined by the number of G418-resistant clones in the dishes (Fig. 1E, F). Then, large-scale parallel DNA transfection and G418 screenings were performed, and with 10 parallel experiments, more than 530,000 G418-resistant clones with PB-GSV integrations were finally obtained.

### Screening for the highly metastatic subclones in vivo to identify the candidate genes

**Fig. 2:**
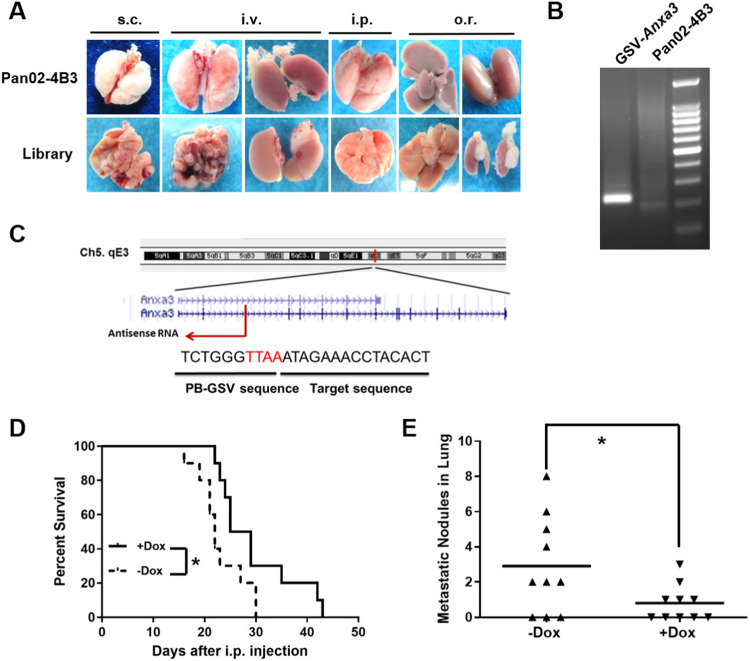
Screening for highly metastatic subclones in vivo to identify the candidate genes. **A** Enhanced metastatic capacity of the mutagenic libraries in vivo, representative images were shown. *Upper:* organs from mice transplanted with control cells Pan02-4B3. *Lower:* organs from mice transplanted with the library. **B** *Anxa3* was identified by Splinkerette PCR from the metastatic lesions. Target bands were extracted and sequenced. **C** Sequence alignment in UCSC Blat for *Anxa3*. PB-GSV initiated an antisense RNA which was opposite to the direction of *Anxa3* transcription. **D** Overall survival time was prolonged when intraperitoneally transplanting GSV-*Anxa3* subclone into mice with Dox treatment (+Dox) as compared to the control group (−Dox) (*n* = 10, *p < 0.05, Kaplan–Meier survival analysis). **E** Macro-metastatic lung nodules decreased when subcutaneously transplanting GSV-*Anxa3* subclone into mice with Dox treatment (+Dox) as compared to the control group (−Dox). Mice were sacrificed on day 55 (*n* = 10, *p < 0.05).

Metastasis is a complex process controlled by multiple genes [61]. To identify the metastasis-controlling genes, Pan02-4B3 cells, and mutant libraries were transplanted into C57BL/6 mice using four different methods, including subcutaneous (s.c.), intraperitoneal (i.p.), orthotopic (o.r.), and intravenous (i.v.) injection. The mutant libraries were divided into 7 small ones to ensure that each PB-GSV integration had enough coverage in the primary tumors. As a result, a total of 168 mouse pancreatic cancer models were established. The injection details were summarized in Supplementary Table S2. The mice were then sacrificed for the examination of metastatic lesions when cachexia and dyspnea developed. It showed that the mice burdened with Pan02-4B3 cells developed only a few metastatic events (Table 1). Mutant libraries showed significantly enhanced metastatic capacity in vivo as compared to the parental Pan02-4B3 cells (Fig. 2A). The metastatic lesions were carefully dissected from lung, liver, brain, adrenal tissues, or others, finally a total of 159 subpopulations of the highly metastatic tumor cells were obtained from the primary culture (summarized in Supplementary Table S3).

**Table 1:** Metastatic events in mouse models transplanted with Pan02-4B3 subclone.

Mouse models	Metastatic events in specific organs			
	Lung	Liver	Brain	Others
s.c.	1/5	0/5	0/5	0/5
i.p.	0/6	0/6	0/6	3/6^a^
i.v.	0/6	0/6	0/6	0/6
o.r.	0/6	2/6	0/6	4/6^a^

Mouse models: s.c. (subcutaneously), i.p. (intraperitoneally), i.v. (intravenously), o.r. (orthotopically).

^a^Tumors invade the portal area, diaphragm, gastrointestinal tract, and tissues surrounding the adrenal and kidney.

To investigate whether tetracycline could reverse the metastatic phenotypes by turning off the PB-GSV mutagenesis, the metastatic subpopulations from each injection method were separately mixed and re-injected into the mice the same way they were obtained. As expected, the number of metastatic nodules decreased significantly in the groups with Dox treatment (+Dox group) as compared to those without Dox treatment (−Dox group) (Fig. S2). All the metastatic nodules from both groups, including those from the organs without visible metastatic nodules from +Dox groups, which could still generate G418-resistant cells in some organs from the primary culture, were separately collected. Finally, a total of 107 highly metastatic subclones were obtained (summarized in Supplementary Table S4). The genes which could reverse the metastatic phenotype by turning off the mutagenesis in vivo were considered the candidate causative genes.

To identify the PB-GSV-targeted sites, genomic DNA was isolated from the subclones and the PB-GSV-flanking regions were amplified using the parallel Splinkerette PCR. The sequence alignment analysis of the amplified fragments using the UCSC Genome Browser and NCBI mouse genome resources identified a total of 46 PB-GSV integration sites in the mouse genome. The PB-GSV integration sites were present in many chromosomes except chromosomes 12, 13, 18, 19, and Y. Among the 46 PB-GSV integration sites, 87% (40/46) were located within or near the known or predicted transcriptional units, especially in the introns (77%). The analysis of integration sites identified a total of 36 candidate genes, including 12 from –Dox group, 17 from +Dox group, and the other 7 from both groups. Additionally, 4 non-coding RNAs (*2610020C07Rik*, *Ptgs2os*, *1700045H11Rik* and *4933432G23Rik*), including one (*1700045H11Rik*) from –Dox group, were also identified (summarized in Supplementary Table S5). The top 5 candidate genes, which were ranked by their mutagenic frequency in all highly metastatic subclones, included *Errfi1, Vmp1, Anxa2, Ywhaz* and *Anxa3* (Table 2). The integration sites of these 5 genes were further validated (Fig. S3). The pathway analysis of the identified genes showed that these genes were mainly involved in 12 functional pathways, including cell-cell adhesion or integrin signaling (*Ywhaz, Vmp1, Anxa2, Fat3, Actb* and *Parvb*), protein or receptor tyrosine kinase (RTK) pathway (*Errfi1, Lrig1, Mucl1* and *Abl2*), apoptosis pathway (*Ywhaz, Anxa3, Aig1, Lrig1, Vmp1, Abl1, Tmem192* and *Cdkn2aip*), regulation of immune response (*Anxa3, Masp1* and *Mucl1*), and others. Six genes (*Mgtpbp6, Plcxd1, Wdr52, Ptgfrn, Rsl1d1, Def8*) and non-coding RNAs, which were mainly identified in the clones from +Dox groups, were of unknown functions in the pancreatic cancer metastasis (summarized in Supplementary Table S6).

**Table 2:** The top 5 candidate genes identified in the highly metastatic subclones.

Genes	Integration sites	Metastatic sites	Frequency	−Dox (*n*, %)	Models
*Errfi1*	First intron	Lung, kidney, brain, bone, skin	26	19 (73.1)	i.v.
*Vmp1*	First intron	Lung, kidney, brain, bone, skin	26	19 (73.1)	i.v.
*Anxa2*	Third intron	Liver, kidney, adrenal, lung, brain	21	7 (33.3)	o.r.
*Ywhaz*	Second intron	Lung, liver, kidney, brain, bone	20	15 (75)	s.c.
*Anxa3*	Second intron	Lung, liver	8	5 (62.5)	i.p.

The candidates were ranked by the frequency in all highly metastatic subclones. “−Dox” indicates the subclones were cultured from the mice without DOX treatment.

To further validate the regulated mutagenic system, the metastatic subclone with PB-GSV-targeted *Anxa3* was transplanted into the mice treated with or without Dox. Splinkerette PCR (Fig. 2B) and sequence alignment analysis (Fig. 2C) showed that PB-GSV integrated into the *Anxa3* intron region. We found that Dox treatment could prolong the animal survival time (Fig. 2D) and significantly reduce the macro-metastatic nodules in lungs (Fig. 2E). These findings indicated the establishment of a reliable regulated high-throughput screening strategy for the identification of candidate genes, which might play causative roles in pancreatic cancer metastasis.

### Functional validation of YWHAZ as a key regulator of cancer metastasis

*Ywhaz* was one of the most frequently PB-GSV-targeted genes. It was targeted 3-times more frequently in the −Dox group (15 clones) with large metastatic lesions as compared to the +Dox group (5 clones) without visible metastatic nodules (Table 2). PB-GSV integration was identified from the subcutaneous mouse models, in which the metastatic process was believed to be much more complicated than other models. Considering the frequency and the ratio of −Dox/+Dox groups, we selected *Ywhaz* for further functional validation.

**Fig. 3:**
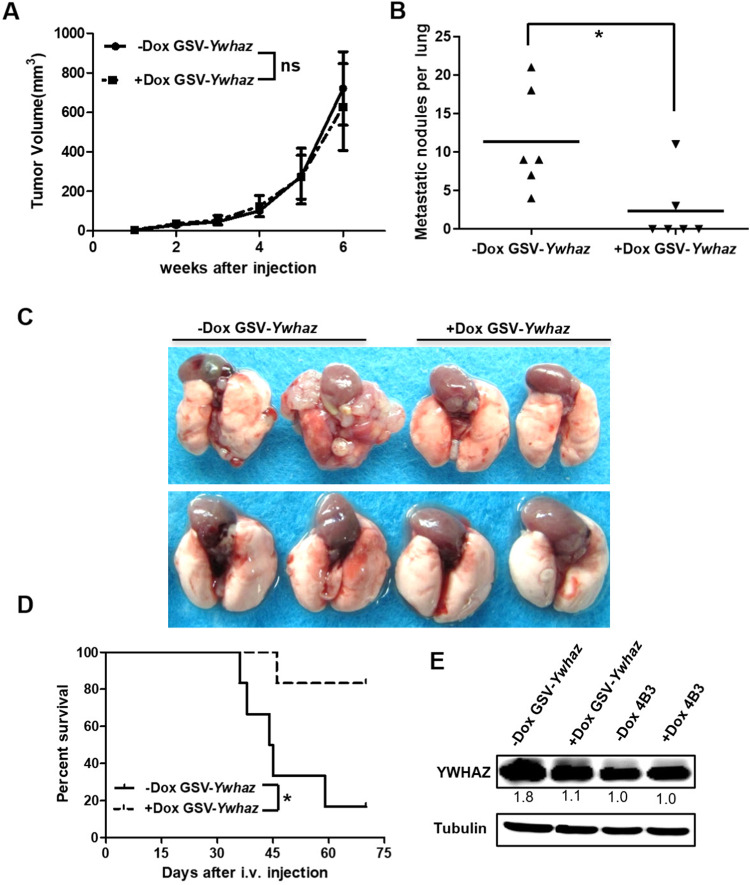
Functional validation of *Ywhaz* as a metastasis-promoting gene. **A** Tumor growth was not affected in mice subcutaneously transplanted with GSV-*Ywhaz* subclones with Dox treatment (*n* = 6; ns, not significant). **B** The total number of macro-metastatic lung nodules decreased with Dox treatment (+Dox) as compared to the control group (−Dox). Mice were sacrificed on day 60 after subcutaneous transplantation of GSV-*Ywhaz* (*n* = 6, *p < 0.05). **C** Representative images of macro-metastatic lung nodules. **D** Overall survival time was prolonged when intravenously transplanting GSV-*Ywhaz* subclone into mice with or without Dox treatment (*n* = 6, *p < 0.05, Kaplan–Meier survival analysis). **E** Western blotting analysis showed the upregulation of YWHAZ expression in GSV-*Ywhaz* subclones and downregulation of YWHAZ expression after DOX treatment.

Dox treatment had no impact on the growth of subcutaneous tumors (Fig. 3A). Immunohistochemical results also showed no significant difference in the proliferation index in −Dox/+Dox groups, as indicated by the Ki67 index (Fig. S4). However, Dox treatment significantly decreased the number of visible metastatic lung nodules when the cells harboring PB-GSV-targeted *Ywhaz* (GSV-*Ywhaz* clone) were subcutaneously injected into the C57BL/6 mice (Fig. 3B and C). Dox treatment could also prolong the overall survival rate of the mice when the GSV-*Ywhaz* clones were injected into the tail vein of C57BL/6 mice (Fig. 3D). The expression level of YWHAZ in GSV-*Ywhaz* clone was increased as compared with the parental Pan02-4B3, which was confirmed by Western blotting (Fig. 3E) and also by immunofluorescence analysis (Fig. S5A). It was reduced to the base level when cells were treated with Dox in vitro as shown in Western blotting (Fig. 3E), or when mice burdened with GSV-*Ywhaz* clone were treated with Dox in vivo as shown in immunochemical analysis with the subcutaneous tumors (Fig. S5B).

**Fig. 4:**
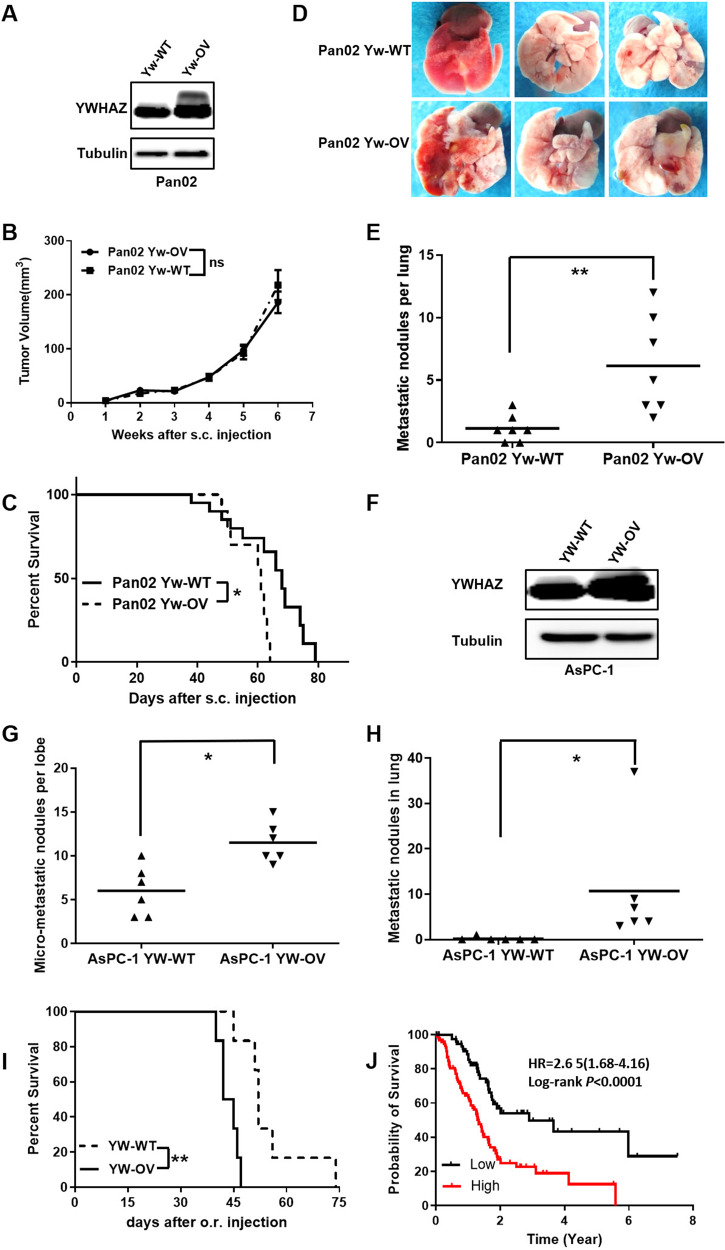
YWHAZ is a regulator of mouse and human pancreatic cancer metastasis. **A** Western blotting analysis showed YWHAZ was overexpressed in Pan02 cells (Pan02 Yw-OV) compared to the control cells (Pan02 Yw-WT). **B** Tumor growth was not affected by YWHAZ overexpression in s.c. model (*n* = 7; ns, not significant). **C** YWHAZ overexpression was associated with worse overall survival in s.c. model (*n* = 12, *p < 0.05, Kaplan–Meier survival analysis). **D** YWHAZ overexpression resulted in more lung macro-metastasis in s.c. model. Representative images of macro-metastatic lung nodules were shown. **E** Macro-metastatic lung nodules were compared between Pan02 Yw-OV and Pan02 Yw-WT (*n* = 7, *p < 0.05). **F** Validation of YWHAZ overexpression in AsPC-1 by Western blotting analysis (AsPC-1 YW-OV). **G** and **H** YWHAZ overexpression resulted in more micro-metastatic (**G**) and macro-metastatic (**H**) lung lesions in o.r. model (*n* = 6, *p < 0.05). **I** Kaplan–Meier survival analysis showed that YWHAZ overexpression significantly shortened the overall survival time in AsPC-1 cells in o.r. model (*n* = 5, *p < 0.01). **J** Kaplan–Meier survival analysis showed that high YWHAZ expression in pancreatic tumor samples was associated with a worse prognosis in clinical patients (data from TCGA and HPA database, *N* = 173, median survival time in high and low expression group was 15.33 and 23.17 months, respectively). The multivariate COX regression analysis, integrated with patients’ age, gender, and TNM stage, revealed YWHAZ was an independent negative prognostic factor for pancreatic cancer (HR = 2.65, 95%; CI:1.68–4.16; *p* < 0.0001).

Next, YWHAZ was overexpressed in the parental Pan02-4B3 cells (Yw-OV) through lentiviral transduction containing mouse *Ywhaz* cDNA (Fig. 4A). As compared to the control group, the overexpression of YWHAZ was associated with the increased number of macro-metastatic lung lesions without affecting the tumor growth in vivo (Fig. 4B, D, E) and also a worse overall survival rate in the subcutaneous mouse models (Fig. 4C). The overexpression of YWHAZ also shortened the overall survival time in the orthotopic mouse models (Fig. S6A). Primary pancreatic tumors were shown in Fig. S6B, which aggressively invaded the nearby organs, like the liver, kidney, and adrenal.

To determine whether YWHAZ played a promoting role in the metastasis of human pancreatic cancer, YWHAZ was overexpressed in AsPC-1 cells (YW-OV) through lentiviral transduction containing human *YWHAZ* cDNA (Fig. 4F). As compared to the control group, the lungs from the mice injected with AsPC-1-YW-OV had not only more micro-metastatic lesions (Figs. 4G and S7) but also more visible macro-metastatic nodules in the orthotopic mouse models (Fig. 4H). The overexpression of YWHAZ in AsPC-1 also significantly shorten the mouse overall survival time in the orthotopic models (Fig. 4I). All these data suggested that YWHAZ was a key regulator in both the mouse and human pancreatic cancer metastasis.

Interestingly, high YWHAZ expression in pancreatic tumor samples was associated with a worse prognosis in clinical patients. Median survival time in the high and low expression groups was 15.33 and 23.17 months, respectively (*p* < 0.0001). YWHAZ expression level was an independent negative prognostic factor for patients with pancreatic adenocarcinoma, with a hazard ratio (HR) of 2.65 (95% confidence interval: 1.68–4.16, *p* < 0.0001) in the multivariate COX regression analysis integrated with variables including age, gender and TMN stage (Fig. 4J). In addition, other candidate genes mainly from −Dox group including ANXA2, ANXA3, CENPE, PARVB, and MUCL1 were also found to be independently associated with the prognosis of patients with pancreatic adenocarcinoma (summarized in Supplementary Table S7). The alteration frequency of the *YWHAZ* gene was about 8% in pancreatic cancer samples, including point mutation and amplification, which was summarized in Supplementary Table S8 along with the other 5 genes with prognostic values.

### YWHAZ enhanced the metastatic phenotypes of pancreatic cancer via EMT

**Fig. 5:**
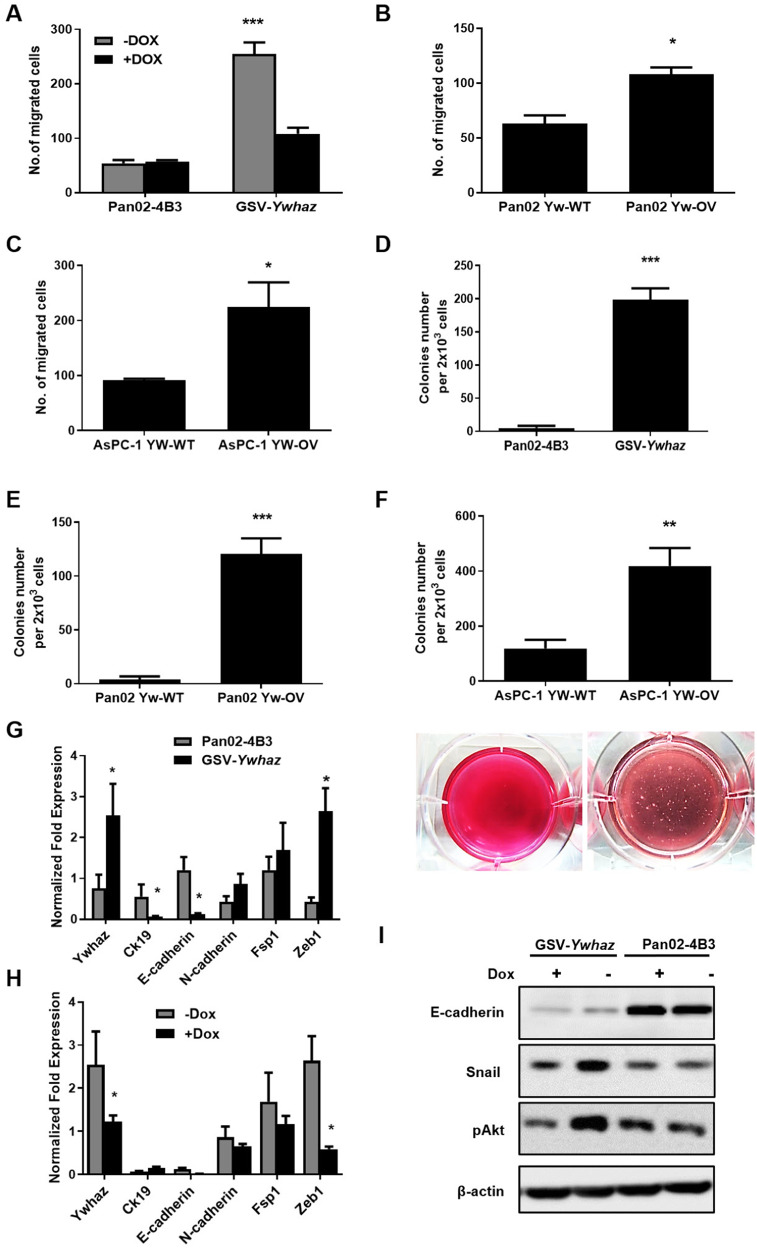
Overexpression of YWHAZ promotes metastatic phenotypes. **A** The GSV-*Ywhaz* subclone showed enhanced invasion capacity as compared to the parental Pan02-4B3 cells with transwell assay (*n* = 3, ***p < 0.0001). **B** YWHAZ overexpression promotes tumor invasion in Pan02 cells with transwell assay (*n* = 3, *p < 0.05). **C** YWHAZ overexpression in AsPC-1 cells resulted in enhanced invasion capacity with transwell assay (*n* = 3, *p < 0.05). **D** The GSV-*Ywhaz* subclone showed enhanced anchorage-independent growth capacity in soft agar as compared to the parental Pan02-4B3 cells (*n* = 5, ***p < 0.0001). **E** YWHAZ overexpression promotes anchorage-independent growth in soft agar in Pan02 cells (n = 6, ***p < 0.0001). **F** YWHAZ overexpression resulted in enhanced anchorage-independent growth capacity in soft agar in AsPC-1 cells. (*n* = 6, ***p < 0.05). The representative image of colonies was shown. **G** The expression of differentiation and EMT-related markers were investigated by qPCR (*n* = 3, *p < 0.05). **H** The expression of differentiation and EMT-related markers in the GSV–*Ywhaz* subclone was compared with or without Dox treatment by qPCR. The expression of mRNA was normalized against *GAPDH* (*n* = 3, *p < 0.05). **I** Western Blotting analysis showed the downregulation of E-cadherin expression, the upregulation of phosphorylated AKT (pAKT), and Snail expression in GSV-*Ywhaz* subclone.

Tumor metastasis is a multistep process, including local invasion, intravasation, transport, extravasation, and colonization. The enhanced invasion capacity of tumor cells is the first step in metastasis in vivo. The transwell invasion assay demonstrated that the GSV integration increased the number of migrated cells, and doxycycline treatment decreased the migration ability of clone GSV-*Ywhaz* (Fig. 5A). Overexpression of YWHAZ in Pan02 and AsPC-1 cells also enhanced the invasion capacity in transwell assay (Fig. 5B, C). Although the overexpression of YWHAZ did not affect the proliferation rate of monolayer cell cultures, it significantly increased their anchorage-independent growth on soft agar (Fig. 5D, E). An increase in the growth of soft agar was also found in the AsPC-1-YW-OV as compared to the AsPC-1-control cells (Fig. 5F).

A key event in promoting stationary tumor cells to migrate and invade is the epithelial–mesenchymal transition (EMT) program, which is characterized by cell morphological changes, a loss of epithelial cell markers, and an upregulation in the expression of mesenchymal cell markers. In cell culture, morphological changes in the GSV-*Ywhaz* clone were observed as spindle-like cells with few branches, which were quite different from those of the epithelial Pan02-4B3 cells (Fig. S5A). These changes were also observed in the YWHAZ-overexpression Panc1 cells (Fig. S8). To evaluate the expression levels of epithelial and mesenchymal marker genes, real-time quantitative PCR was performed. We found that the GSV-*Ywhaz* clone expressed lower levels of the epithelial markers, such as *Ck19* and *E-cadherin*, and higher levels of the mesenchymal marker gene, like *N-cadherin*, as compared to the parental Pan02-4B3 cells (Fig. 5G).

To confirm that the epithelial cancer cells could activate the mesenchymal transition at the transcriptional level, transcription factors fibroblast-specific protein 1 (Fsp1) and zinc finger E-box-binding homeobox (Zeb1) were quantified. We found that Zeb1 was highly expressed in the GSV-*Ywhaz* clone as compared to the parental Pan02-4B3 cells (Fig. 5G). The expression of Zeb1 returned to the parental level, along with the YWHAZ expression, when treating the GSV-*Ywhaz* clones with Dox to turn off the mutagenesis effect (Fig. 5H). The E-cadherin protein level was dramatically decreased in the GSV-*Ywhaz* clones and was not reversed by Dox treatment. However, EMT-related transcription factor Snail was highly expressed in the GSV-*Ywhaz* clones and could be reversed by Dox treatment. The phenomenon was also observed in phosphorylated AKT expression, which was a classic downstream-activated protein of YWHAZ (Fig. 5I).

**Fig. 6:**
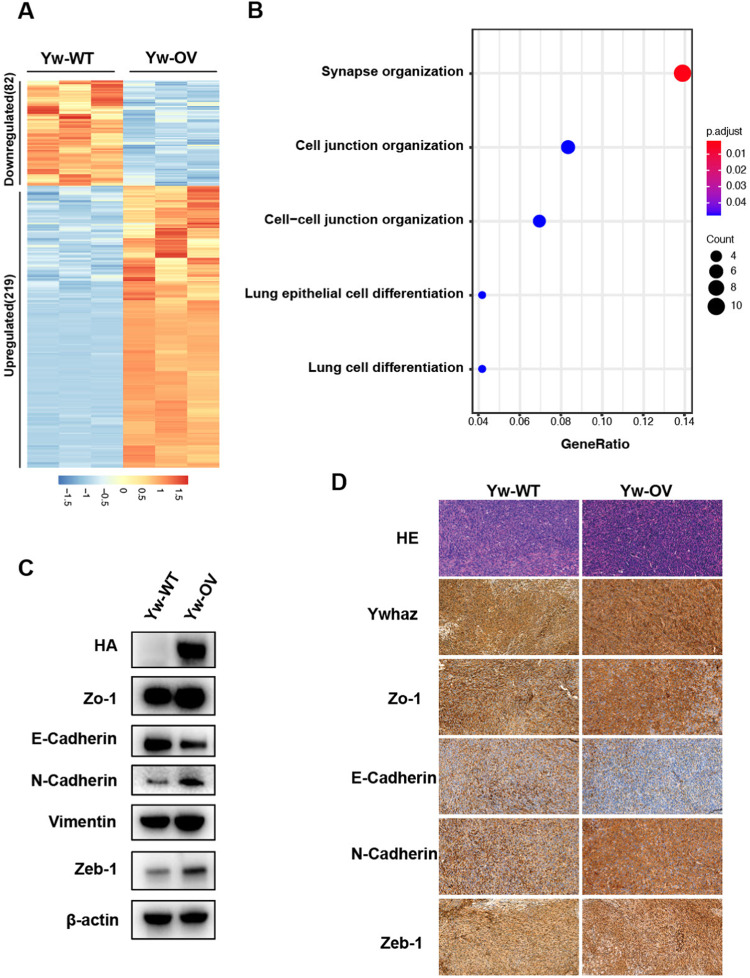
YWHAZ promotes pancreatic cancer metastasis via EMT. **A** Heat map of the DEGs identified by RNA-seq in Pan02 control cells (Yw-WT) and YWHAZ overexpression cells (Yw-OV) (>2-fold, *p* < 0.05, *n* = 3). **B** Top enriched GO terms in downregulated genes. **C** Western blot analysis of the EMT biomarkers and transcription factors, *Ywhaz* was cloned with a fusion HA-tag and anti-HA shows the expression of YWHAZ. **D** Hematoxylin-eosin staining and immunohistochemical staining of the EMT biomarkers and transcription factors in subcutaneous tumors. E-cadherin and ZO-1 not only showed a difference in intensity but also a change in location after YWHAZ overexpression (*n* = 3, *p* < 0.05).

To further decipher the mechanism behind YWHAZ-mediated metastasis, we performed RNA-seq in Pan02 control (Yw-WT) and YWHAZ overexpression cells (Yw-OV). We identified 219 and 82 significantly upregulated or downregulated genes in Yw-OV relative to Yw-WT cells (≥ 2-fold, *p*-value ≤ 0.05, Fig. 6A). Interestingly, functional annotation of the downregulated genes by GO analysis mainly identified cell-cell junction organization pathway (Fig. 6B), which is the key event in EMT. Moreover, gene set enrichment analysis (GSEA) also revealed that YWHAZ was associated with EMT-related pathways, including “focal adhesion” and “ECM receptor interaction” (Fig. S9A), further supporting the potential effects of YWHAZ on EMT. Importantly, consistent with the RNA-seq results, Western blot analysis for EMT markers also revealed that Yw-OV cells harbored EMT features. Epithelial phenotype markers E-cadherin decreased, while mesenchymal phenotype-associated molecules, ZO-1, N-cadherin, Vimentin, and ZEB-1 were all upregulated in Yw-OV cells (Fig. 6C), similar results were also observed in the subcutaneous tumors (Fig. 6D) and metastatic lung tumors (Fig. S10) with immunohistochemistry analysis. These results indicated that the enhanced metastatic phenotypes mediated by the overexpression of YWHAZ result from its effects on regulating the epithelial-to-mesenchymal transition-related genes and pathways.

## Discussion

A major goal of research in cancer biology is to understand the puzzling complexity of cancer characteristics, which distinguish them from benign lesions and enable tumor growth and dissemination of metastasis. The basic six hallmarks of cancer are (1) sustaining proliferative signaling, (2) evading growth suppressors, (3) resisting cell death, (4) inducing angiogenesis, (5) enabling replicative immortality, and (6) activating invasion and metastasis [62]. Similarly, the process of cancer metastasis consists of the following steps: primary tumor growth and angiogenesis, invasion into the circulatory system, migration out of the system, and colonization in the distant organs. The completion of these steps is dependent on both the intrinsic capabilities of cancer cells and the tissue-specific microenvironment [61]. The functional capabilities of the cancer cells to proliferate, survive, and disseminate are acquired for long-term multistep tumorigenesis. This results in genomic instability in cancer cells and causes inflammation in the form of premalignant or malignant lesions due to the response of the immune system in the surrounding environment [62]. This study attempted to evaluate the pathogenic mechanism of pancreatic cancer metastasis and identify the causative genes. A high-throughput mutagenic library was established in a low-metastatic mouse pancreatic cancer cell line to obtain the loss-of-function or gain-of-function genes and long non-coding RNAs among others. Furthermore, the highly metastatic phenotypes were screened and validated using the mouse pancreatic cancer models with competent immune responses.

The successful screening can be determined by its ability to directly relate mutation with the aimed phenotypes. This requires saturated mutations in the genome, which can be achieved by a mutagen, and the absence of the selected phenotypes. The genomic screen system developed in this study had the advantage of the high integration efficacy of the engineered gene search vector based on PB-GSV and tetracycline-inducible mutagenesis. The system could efficiently reverse the metastatic phenotypes by turning off the mutagenic effects to rule out possible passenger mutations or gain-of-adaption by spontaneous mutations during the screening process. Consistent with the previous studies [41, 42], this study showed that PB-GSV, when co-transfected with a separate transposase, could efficiently integrate into the tetranucleotide TTAA sequence in the genome, preferably into the transcriptional sites (85%), especially in the introns (77%). In the first screening, more metastasis was observed in the mice burdened with certain mutagenic libraries as compared to the parental control cells, especially in the s.c and i.v groups. These results were validated by turning off the mutagenesis with Dox treatment in vivo, which reversed the severity of organ metastasis. The primary culture cells generated G418-resistant clones, which were the cells from the micro-metastasis or the cells with gain-of-adaption in the organs as reported in the previous study, in which the highly metastatic subclones of Pan02-P7 were generated [54]. Two genes, *Ywhaz* and *Anxa3*, targeted by the PB-GSV were further studied to confirm their regulatory effects on the mutagenic system. In both cases, Dox treatment not only reduced lung metastasis but also prolonged the survival rate of the mice. The promoting role of YWHAZ was validated in mouse Pan02-4B3 cells and human AsPC-1cells. All these findings demonstrated that the regulated high-throughput mutagenic system based on *piggyBac* is an efficient tool for the discovery of causative genes in cancer metastasis.

YWHAZ, belonging to the 14-3-3 protein family, is a hub protein, which is involved in many signal transduction pathways as well as plays an important role in tumor progression [63]. Numerous studies have demonstrated the frequent up-regulation of YWHAZ and its participation in a wide range of cellular activities, including cell growth, cell cycle, apoptosis, and migration/invasion in multiple types of cancers, such as hepatocellular carcinoma, colorectal cancer, lung cancer, and breast cancer [63–68]. The current study functionally validated *YWHAZ* as a causative gene for metastasis. The overexpression of YWHAZ decreased the survival rate of mice and increased the metastatic phenotypes in both the human and mouse pancreatic cancer cell lines. The overexpression of YWHAZ also resulted in highly aggressive metastatic phenotypes in vitro. The *YWHAZ*-targeted metastatic clones increased the expression levels of the EMT-related genes and transcription factors, suggesting that YWHAZ might regulate metastasis by targeting the EMT-related genes and pathways. YWHAZ has been reported to either stabilize the β-catenin of the Wnt signaling pathway in lung cancer or TbRI in the TGF-β signaling pathway in breast cancer to upregulate the EMT-related genes [65, 66]. Altogether, these data suggested that the functional genomic screens in mouse models based on *piggyBac* transposon provide a novel platform for identifying the genetic determinants of cancer metastasis.

Among the 36 genes identified in this study, 16 genes, including *Ywhaz, Masp1, Lars2, Actb, Cenpe*, and *2610507B11Rik* with gain-of-function mutation, 10 genes, including *Errfi1, Vmp1, Anxa3, E230008N13Rik, Mllt10, Aig1, Parvb, Lrig1, Mucl1* and *Slc25a26* with loss-of-function mutations, were mainly identified in the metastatic subclones with the onset of transposon-mediated mutagenesis, suggesting their causative roles in the pancreatic cancer metastasis. Among them, several genes were identified for the first time as the causative genes for pancreatic cancer metastasis, although some of these genes were previously reported as tumor suppressor genes or oncogenes in certain tumor types. Recently, the perturbation of negative-feedback mechanisms, attenuating the proliferative signaling, was reported as an emerging hallmark of cancer and often regulated by the tumor suppressor genes, such as *ERRFI1* and *LRIG1* [62, 69]. *Errfi1* (ErbB receptor feedback inhibitor 1, also known as *MIG6*) was identified as one of the most frequently mutated genes in this study and is a negative regulator of EGFR signaling [70]. Many studies have shown its down-regulation in tumors, such as glioblastoma multiforme [71], papillary thyroid cancer [72], hepatocellular carcinoma [73], and lung carcinoma [74]. The inhibition of the *Errfi1* gene in mice could induce cancers in the lung, gall bladder, gastrointestinal tract, and bile duct [75]. The inhibition of both the *Errfi1* and *Pten* genes could result in the development and progression of endometrial cancer in a transgenic mouse model [76]. *LRIG1* (leucine-rich and immunoglobulin-like domain 1), which acts as a negative regulator of the EGFR signaling by antagonizing the activity of receptor tyrosine kinases [77, 78] and intestinal stem cell markers [79, 80], plays an important tumor-suppressive role in many cancer types, including brain, breast, skin, kidney, colon, and bladder cancers [81–87]. Bioinformatics analysis-based study revealed that the decreased expression level of LRIG1 was directly correlated with the poor survival outcomes of numerous various tumors, such as bladder, breast, lung, melanoma, and glioma [88]. *LRIG1* was the most frequently disrupted gene, progressing the intestinal adenomas to advanced carcinoma demonstrated in an SB transposon-mediated system, targeting and activating the *KRAS* gene [79]. Most importantly, the soluble LRIG1 could inhibit the growth of glioblastoma in a clinically relevant mouse model independent of the EGFR status [89].

The first step in metastasis is to detach from the primary tumor site and invade the neighboring vascular system, indicating that the disruption of their cell-to-cell adhesion and increased cell migration are the crucial hallmarks of cancer metastasis. Several studies have demonstrated the importance of ACTB in cellular migration [90, 91], and a study exploring the gene profiling of a single metastatic cell also demonstrated its similar role [92]. The actin cytoskeleton dynamics had many regulators, such as the Arp2/3 complex, cofilin, and cortactin, which were overexpressed in cancer and promoted cancer progression (reviewed in [91]). *PARVB*, encoding a focal adhesion protein, could negatively regulate the integrin-linked kinase pathway [93] and was downregulated in breast cancer [94, 95], colorectal cancer [96], and urothelial cell carcinoma [97]. The regulation of cytoskeletal machinery by either *ACTB* gain-of-function or *PARVB* loss-of-function might play causative roles in pancreatic cancer metastasis. On the other hand, the VMP1 expression, which is involved in cell–cell adhesion [98] and the autophagy pathway [99, 100], decreased and closely correlated with the high invasion and metastasis of hepatocellular carcinoma [101] and kidney cancer [98]. The VMP1-related autophagy pathway was identified as either a cellular protective response of acinar cells in the pancreatitis mouse models [99] or a gemcitabine-induced pro-death response in a pancreatic cancer cell line [102]. The overexpression of VMP1 could increase resistance to anti-tumor drugs independent of the autophagic-related effects in pancreatic cancer cell line and mouse models of pancreatic cancer, it was also found to be associated with the grading of pancreatic cancer, especially with the poorly differentiated subtype, but there has been no significant evidence of its role in cancer metastasis [103]. *VMP1* loss-of-function, which accelerated the pancreatic cancer metastasis, might result from quite different pathways, The role of these candidate genes, which are summarized in Supplementary Table S5, should be worthy of future investigation. They might provide new insights into pancreatic cancer metastasis and novel therapeutic strategy for the treatment of pancreatic cancer.

Our system could efficiently mutate the genome, and more importantly, reverse the metastatic phenotypes by turning off the mutagenic effects to rule out possible passenger mutations or gain-of-adaption by spontaneous mutations during the screening process, thus providing a robust screen platform to identify genes causally involved in metastasis. However, there are a few noteworthy limitations to this study. First, the *piggyBac* transposon and transposase are delivered by plasmid transfection, which is inefficient and a barrier to the therapeutic application, the potential utility of viral vectors, like adeno-associated virus or lentivirus, to deliver transposon components could be a better way. Second, *piggyBac* transposon has some insertion site hotspots, which makes the construction of the unbiased genome-wide library more difficult than RNAi or CRISPR. Third, during the screening, the metastatic lesions were removed and cultured, we may lose some metastatic clones due to their growth disadvantage. Fourth, we estimated the genomic coverage of the libraries by Splinkerette-PCR, considering the variations between libraries, high-throughput next-generation sequencing (NGS) technologies may provide a further level of quality control. Fifth, functional annotation of the differentially expressed genes in YWHAZ overexpression cells by GO analysis not only identified EMT-related pathways but also virus response, innate immune response, and other pathways (Fig. S9B), our study did not exclude the possibility that YWHAZ promotes metastasis by other pathways.

In summary, a regulated insertional mutagenesis system based on *piggyBac* transposon was developed to functionally screen the causative genes in mouse pancreatic cancer metastasis in vivo. We identified a total of 16 genes and one long non-coding RNA, which was directly related to the metastatic phenotypes. Some of them have been reported to be involved in metastasis, but not others. The role of YWHAZ in pancreatic cancer metastasis was functionally validated both in vitro and in vivo. These findings suggested that the regulated functional genomic screen was an effective tool to identify the causative genes in cancer metastasis. These identified genes might provide novel insights into the underlying mechanisms of cancer metastasis and the development of a novel therapeutic strategy for the treatment of pancreatic cancer.

## Supplementary information

Supplementary data

Original Data File

Response to the author list changes

## Data Availability

The data supporting this study are available on request from the corresponding author.
