# Supplementary material for: High-throughput functional screen identifies YWHAZ as a key regulator of pancreatic cancer metastasis
Source: Cell Death Dis. 2023 Jul 14;14(7):431. doi: 10.1038/s41419-023-05951-5 (PMC10349114; doi:10.1038/s41419-023-05951-5)
Supplement: Supplementary file 1 — Supplementary data [file 41419_2023_5951_MOESM1_ESM.pdf]

Supplementary Fig S1. Construction of a well-controlled Tet-off system in Pan02 cell line.

A. The activity of the luciferase reporter was regulated by Dox treatment in Pan02-4B3 subclone infected with the pUHC-TRE-luciferase vector containing a modified tetracycline regulated element (TRE). Results are mean ± SEM (n=3; ** $p<0.01$). **B.** pLV-TRE-EGFP vector contained a modified TRE element and a GFP reporter. **C.** GFP expression significantly decreased by Dox treatment in Pan02-4B3 infected with pLV-TRE-GFP, but not the parental Pan02 cells. Scale bar, 100 μm. **D.** Immunochemical analysis to show GFP expression was significantly reduced in the tumor samples derived from the orthotopic mouse models transplanted with Pan02-4B3-GFP when treated with Dox (2mg/ml, 1% sucrose in drinking water, n=5). White scale bar, 500 μm; Black scale bar, 200 μm.

Supplementary Fig S2. The number of metastatic nodules decreased after Dox treatment *in vivo*.

A. For functional validation, the subclones cultured from the first-round screening were separately mixed and re-injected into mice the same way as they were obtained. Gross organs from both groups (-Dox/+Dox) were shown. Left, i.p. (n=10). Right, s.c. (n=10). The arrow indicated metastatic nodules. **B.** The distribution of subclones cultured from metastatic nodules in different mouse models with or without Dox treatment. Although there were no visible metastatic lesions in some mouse models from the +Dox group, G418 resistant subclones could still be cultured. (* $p<0.05$).

Supplementary Fig S3. Validation of PB-GSV targeted *Anxa3* and *Ywhaz* through genomic PCR.

A. The technical principles are illustrated. Primers were designed for specific genes identified with Splinkerette PCR, each of which had a primer targeting either 3'TR or 5'TR on PB-GSV, and the other one was designed within the targeted genes. Products from the other allele could serve as a positive control. **B.** GSV-*Anxa3* integrations were confirmed both in 3'TR and 5'TR of PB-GSV. Results from multiple clones were shown. **C.** GSV-*Ywhaz* integrations were confirmed both in 3'TR and 5'TR of PB-GSV. Each column represents an individual metastatic subclone in B and C. M indicates DNA marker. **D.** *Ywhaz* was identified by Splinkerette PCR from the metastatic lesions. Target band from Splinkerette PCR were extracted and sequenced.

Supplementary Fig S4. GSV-*Ywhaz* integration doesn't affect the proliferation rate of subcutaneous tumors

Hematoxylin-eosin staining and immunohistochemical staining images of the subcutaneous tumors from mouse models injected with GSV-*Ywhaz* clone. No significant difference was found for the immunohistochemical staining of Ki67.

Supplementary Fig S5. Upregulation of YWHAZ expression in GSV-*Ywhaz* clones and derived subcutaneous tumors.

A. GSV-*Ywhaz* mediated YWHAZ upregulation in Pan02. GSV-*Ywhaz* clone was thin spindle shaped, while Pan02-4B3 was polygonal epithelioid. White scale bar, 10 μm; Blue scale bar, 5 μm. **B.** GSV-*Ywhaz* mediated YWHAZ upregulation in subcutaneous tumors.

Supplementary Fig S6. YWHAZ overexpression was associated with poor survival in Pan02

orthotopic mouse model.

A. Kaplan–Meier survival analysis showed that YWHAZ overexpression significantly shortened the overall survival time in Pan02 orthotopic mouse model. (n=8, p=0.015). **B.** Representative images of the pancreatic primary tumors, tumor foci in livers or kidneys were also shown.

Supplementary Fig S7. YWHAZ overexpression significantly increases the number of micro-metastatic lung lesions in AsPC-1 orthotopic mouse models.

A, B. Representative images of the metastatic lesions from orthotopic mouse models. The lesions less than 1mm in diameter was defined as micro-metastasis. **C, D.** Micro-metastasis was measured and confirmed at higher magnification.

Supplementary Fig S8. The morphological changes in YWHAZ overexpressed Panc1 cells.

A. Western blotting analysis confirmed the overexpression of YWHAZ in Panc1 cells. **B.** Panc1 YW-WT cells were polygonal epithelioid, and Panc1 YW-OV cells showed spindle shape. Scale bar, 10 μm.

Supplementary Fig S9. Gene set enrichment analysis and Gene Ontology functional annotation of trait-related genes.

A. Gene Set Enrichment Analysis (GSEA) according to YWHAZ expression level in Pan02 cells.
B. Top enriched GO terms in upregulated genes identified by RNA-seq in Pan02 control cells (Yw-WT) and YWHAZ overexpression cells (Yw-OV).

Supplementary Fig S10. Hematoxylin-eosin staining and immunohistochemical staining of the EMT biomarkers and transcription factors in lung metastatic tumors. YWHAZ, N-Cadherin and ZEB1 showed high expression in YWHAZ overexpressed group and low expression in the control Pan02-4B3 (n=3, $p<0.05$). E-Cadherin and ZO-1 mainly showed location differences from the membrane into the cytoplasm when YWHAZ overexpressed. E-cadherin also showed changes in the intensity of the staining, from moderate to weak positivity (n=3, $p<0.05$).

Figure S1

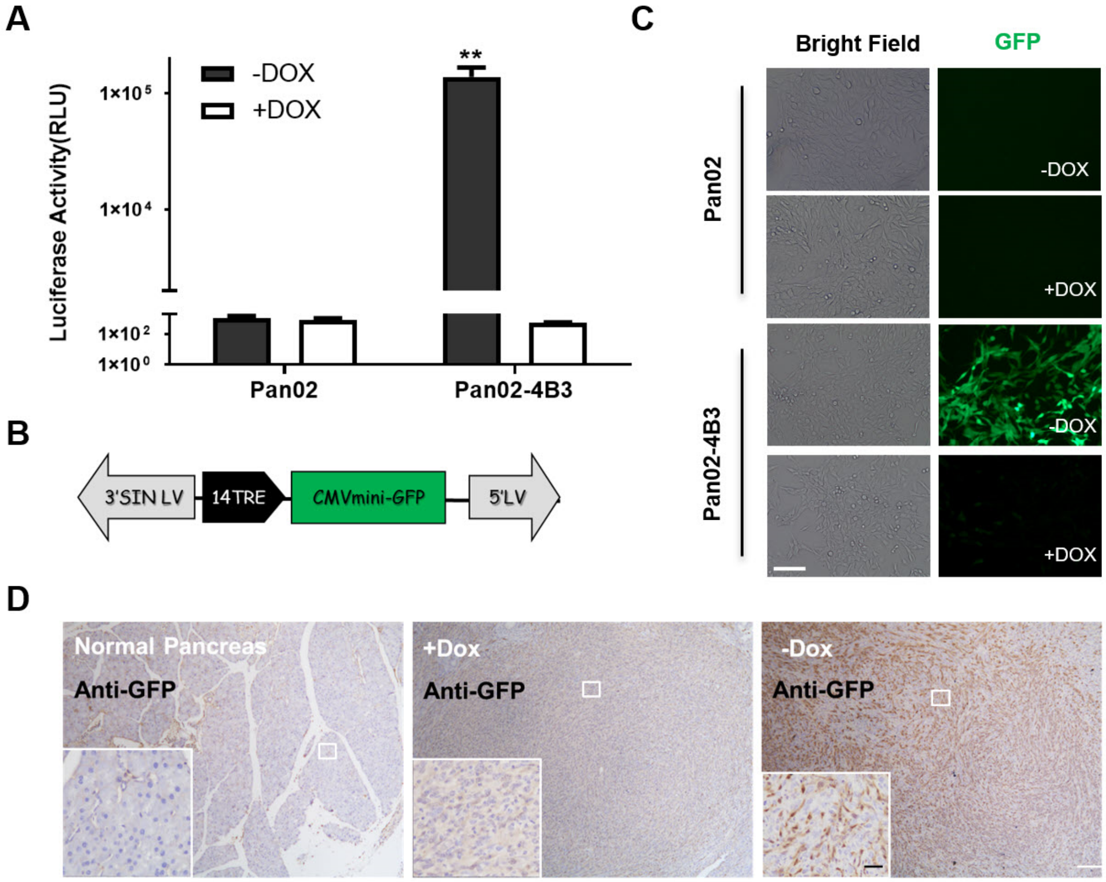
A
Luciferase Activity(RLU)
1×10⁵
1×10⁴
1×10²
1×10⁰
-DOX
+DOX
**
Pan02
Pan02-4B3
B
3'SIN LV
14TRE
CMVmini-GFP
5'LV
C
Bright Field
GFP
Pan02
Pan02-4B3
-DOX
+DOX
-DOX
+DOX
D
Normal Pancreas
Anti-GFP
+Dox
Anti-GFP
-Dox
Anti-GFP

Figure S2

A

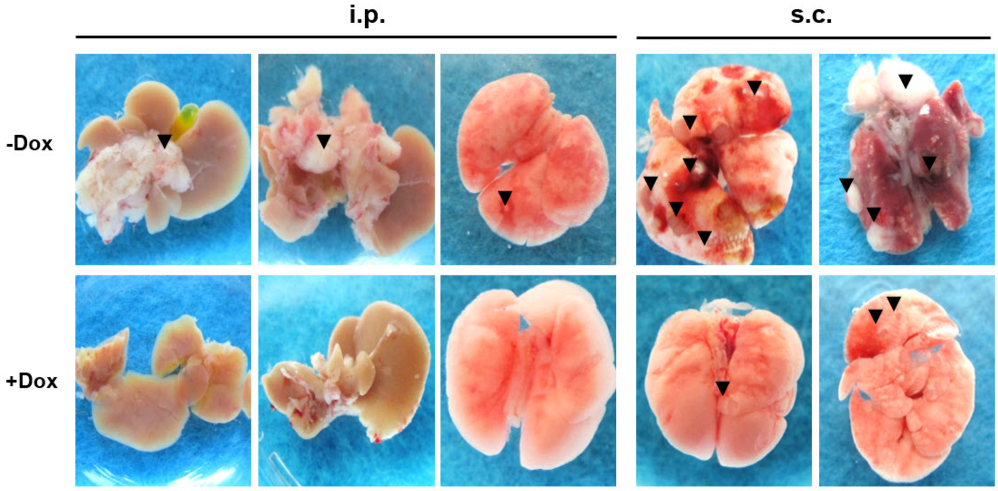

i.p.
s.c.
-Dox
+Dox

B

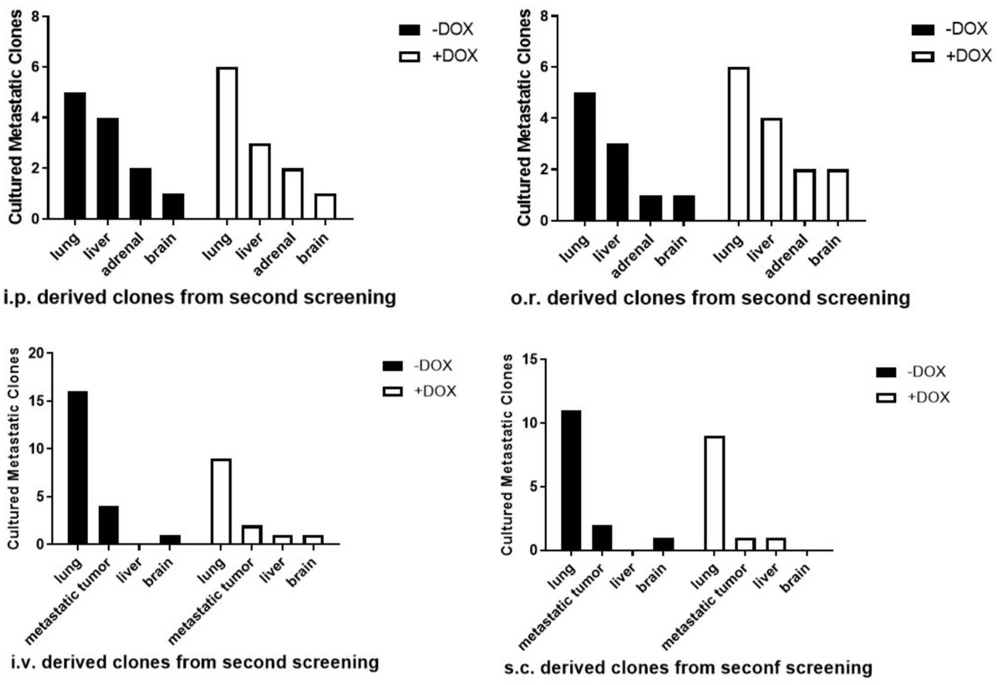

Cultured Metastatic Clones
-DOX
+DOX
lung
liver
adrenal
brain
i.p. derived clones from second screening
o.r. derived clones from second screening
metastatic tumor
i.v. derived clones from second screening
s.c. derived clones from seconf screening

Figure S3

A

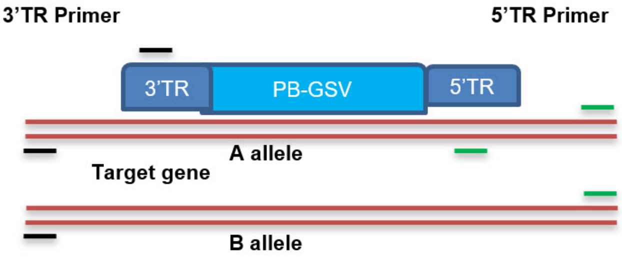

B Validation of GSV-*Anxa3* integrations through PCR in individual metastatic subclones

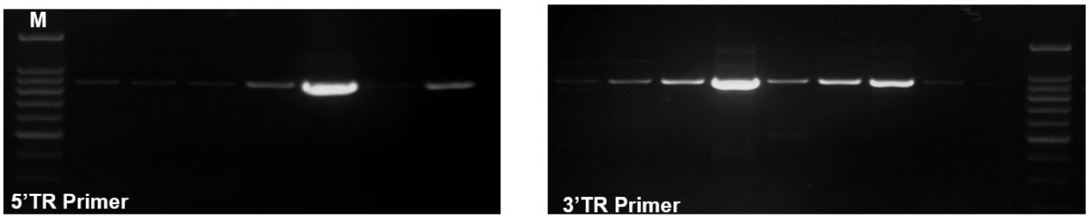

C Validation of GSV-*Ywhaz* integrations through PCR in individual metastatic subclones

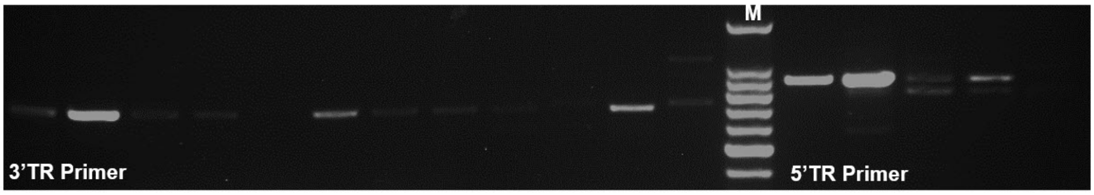

D

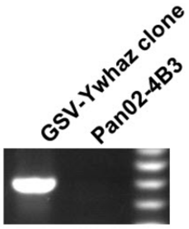

Figure S4

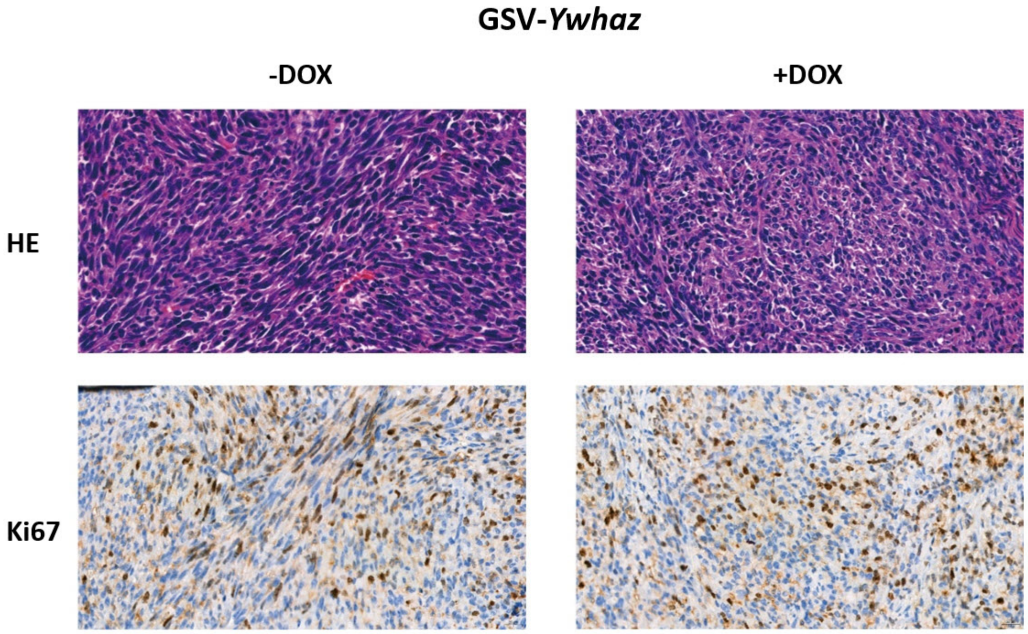

Figure S5

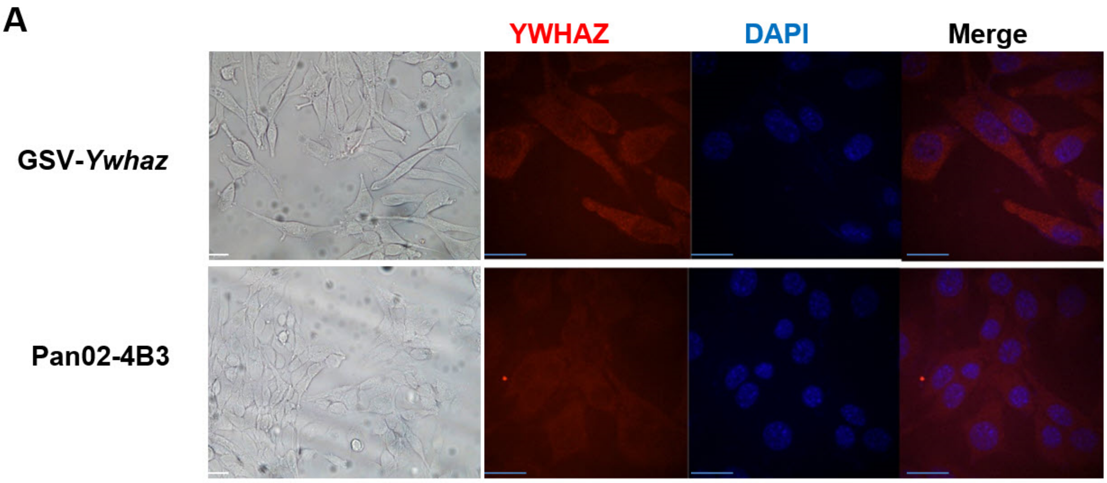
A
YWHAZ
DAPI
Merge
GSV-*Ywhaz*
Pan02-4B3

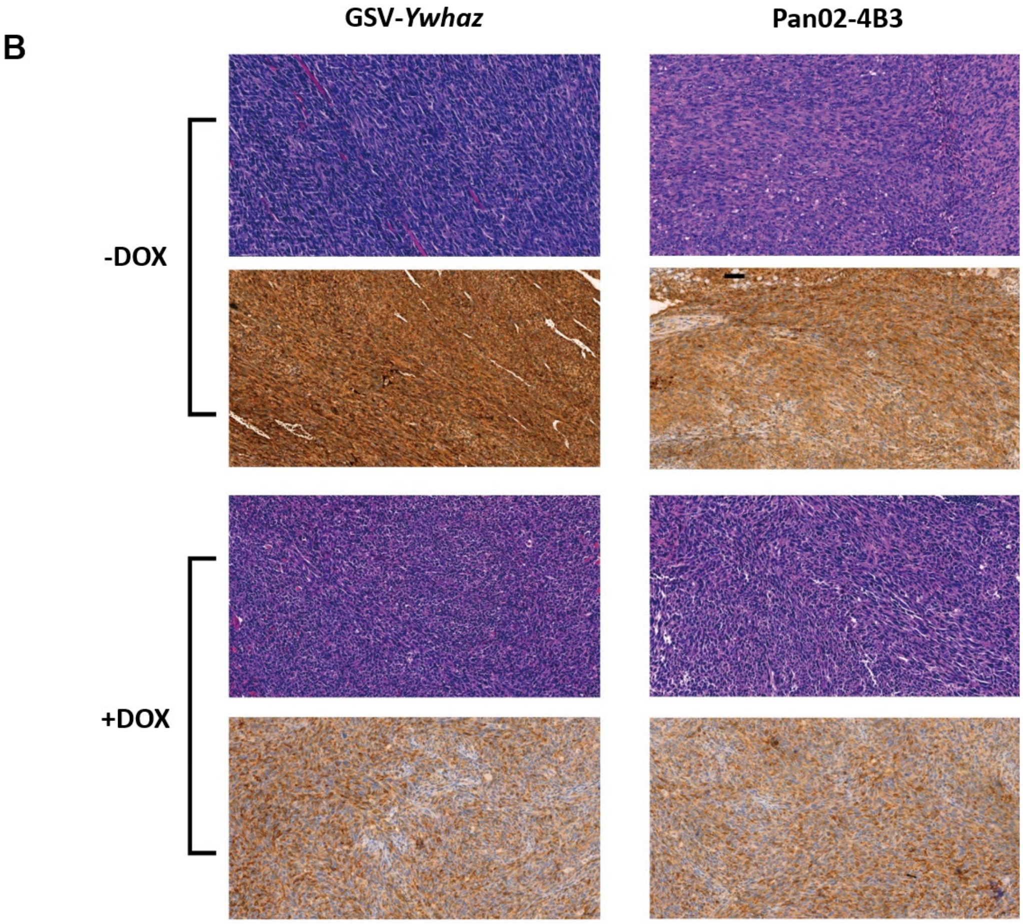
B
GSV-*Ywhaz*
Pan02-4B3
-DOX
+DOX

Figure S6

A

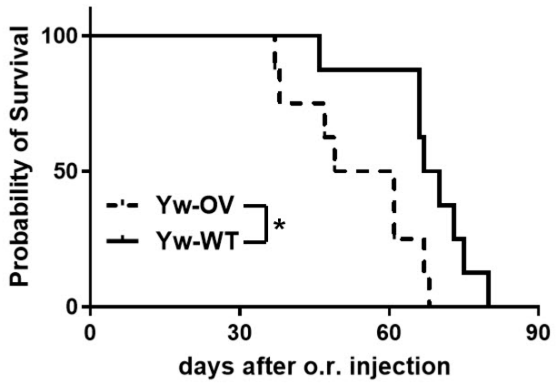

Probability of Survival
100
50
0
0
30
60
90
days after o.r. injection
Yw-OV
Yw-WT
*

B

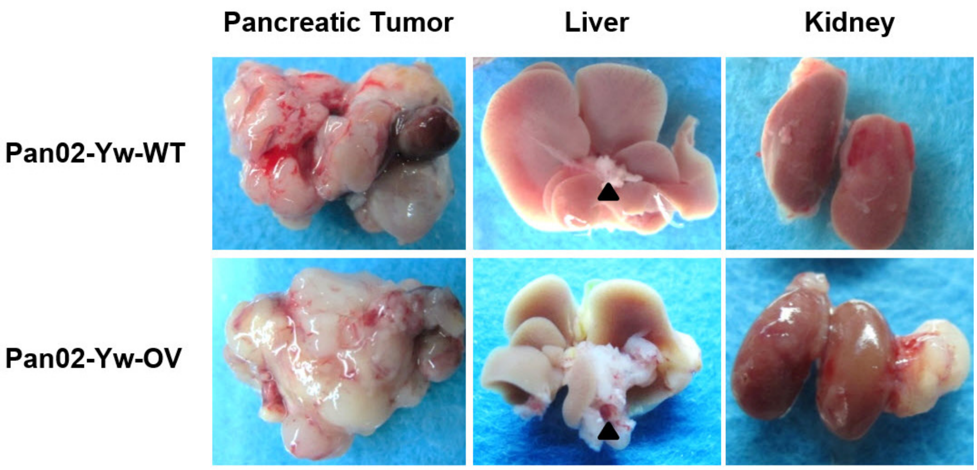

Pancreatic Tumor
Liver
Kidney
Pan02-Yw-WT
Pan02-Yw-OV

Figure S7

A

AsPC-1 YW-WT

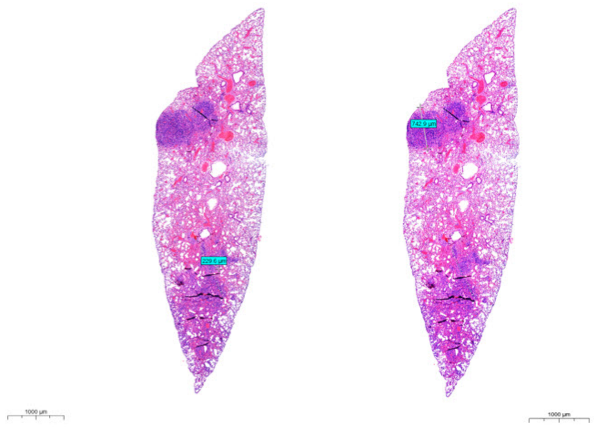

B

AsPC-1 YW-OV

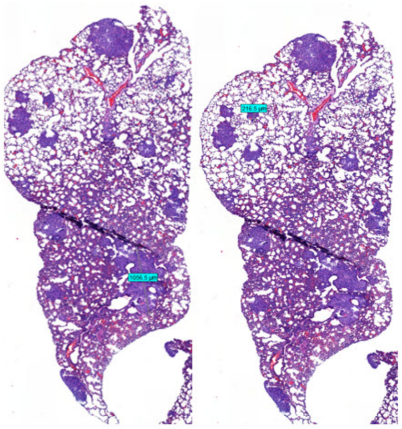

C

AsPC-1 YW-WT

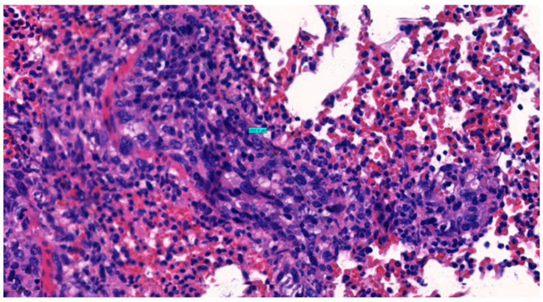

D

AsPC-1 YW-OV

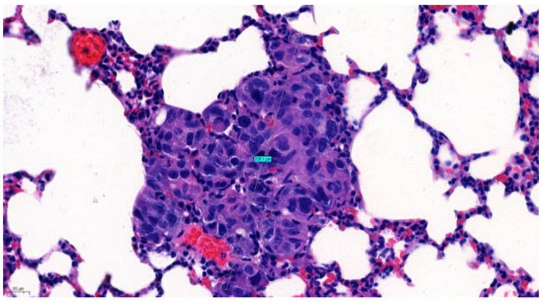

Figure S8

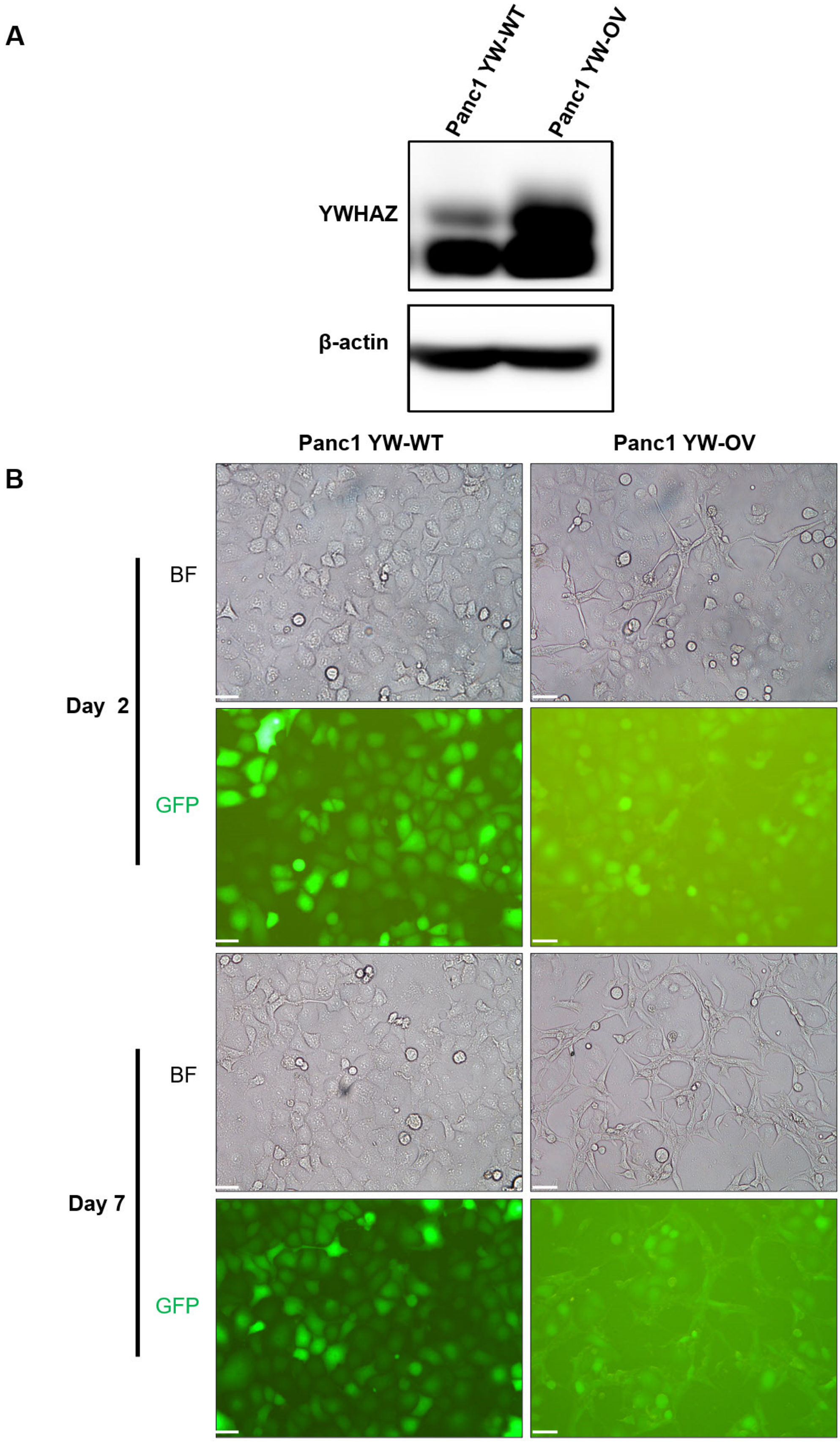
A
Panc1 YW-WT
Panc1 YW-OV
YWHAZ
β-actin
B
Panc1 YW-WT
Panc1 YW-OV
BF
GFP
Day 2
BF
GFP
Day 7

Figure S9

A

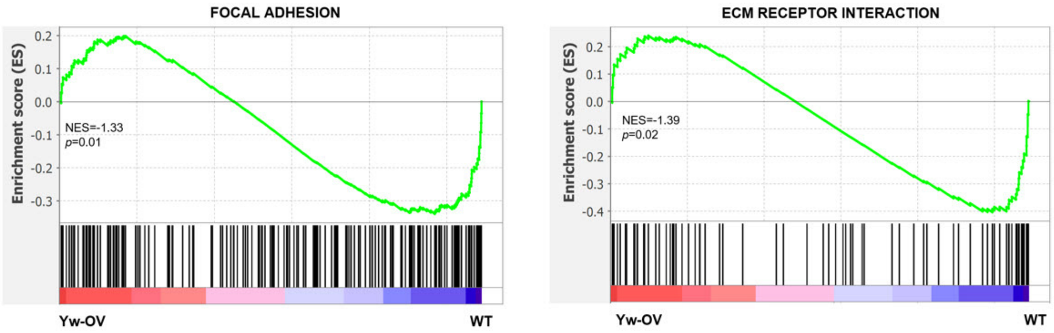

FOCAL ADHESION
Enrichment score (ES)
0.2
0.1
0.0
-0.1
-0.2
-0.3
NES=-1.33
p=0.01
Yw-OV
WT
ECM RECEPTOR INTERACTION
Enrichment score (ES)
0.2
0.1
0.0
-0.1
-0.2
-0.3
-0.4
NES=-1.39
p=0.02
Yw-OV
WT

B

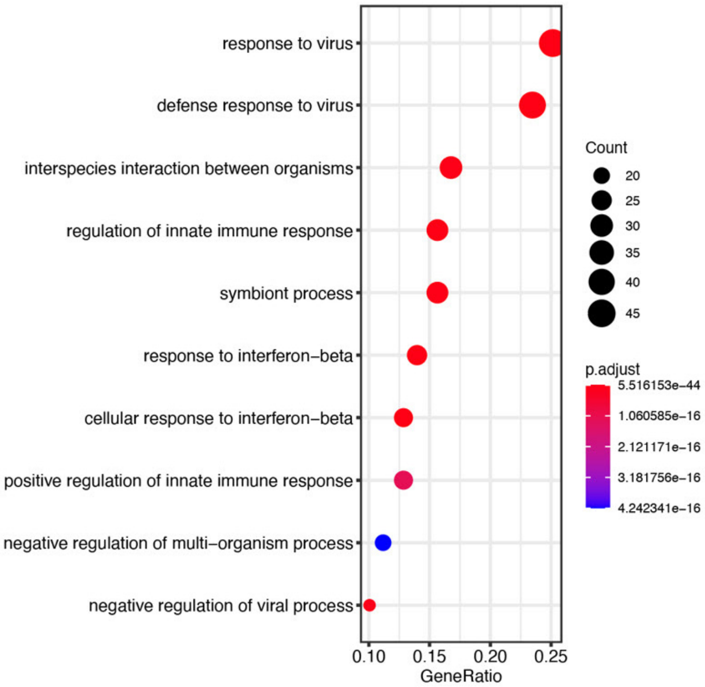

response to virus
defense response to virus
interspecies interaction between organisms
regulation of innate immune response
symbiont process
response to interferon−beta
cellular response to interferon−beta
positive regulation of innate immune response
negative regulation of multi−organism process
negative regulation of viral process
0.10
0.15
0.20
0.25
GeneRatio
Count
20
25
30
35
40
45
p.adjust
5.516153e−44
1.060585e−16
2.121171e−16
3.181756e−16
4.242341e−16

Figure S10

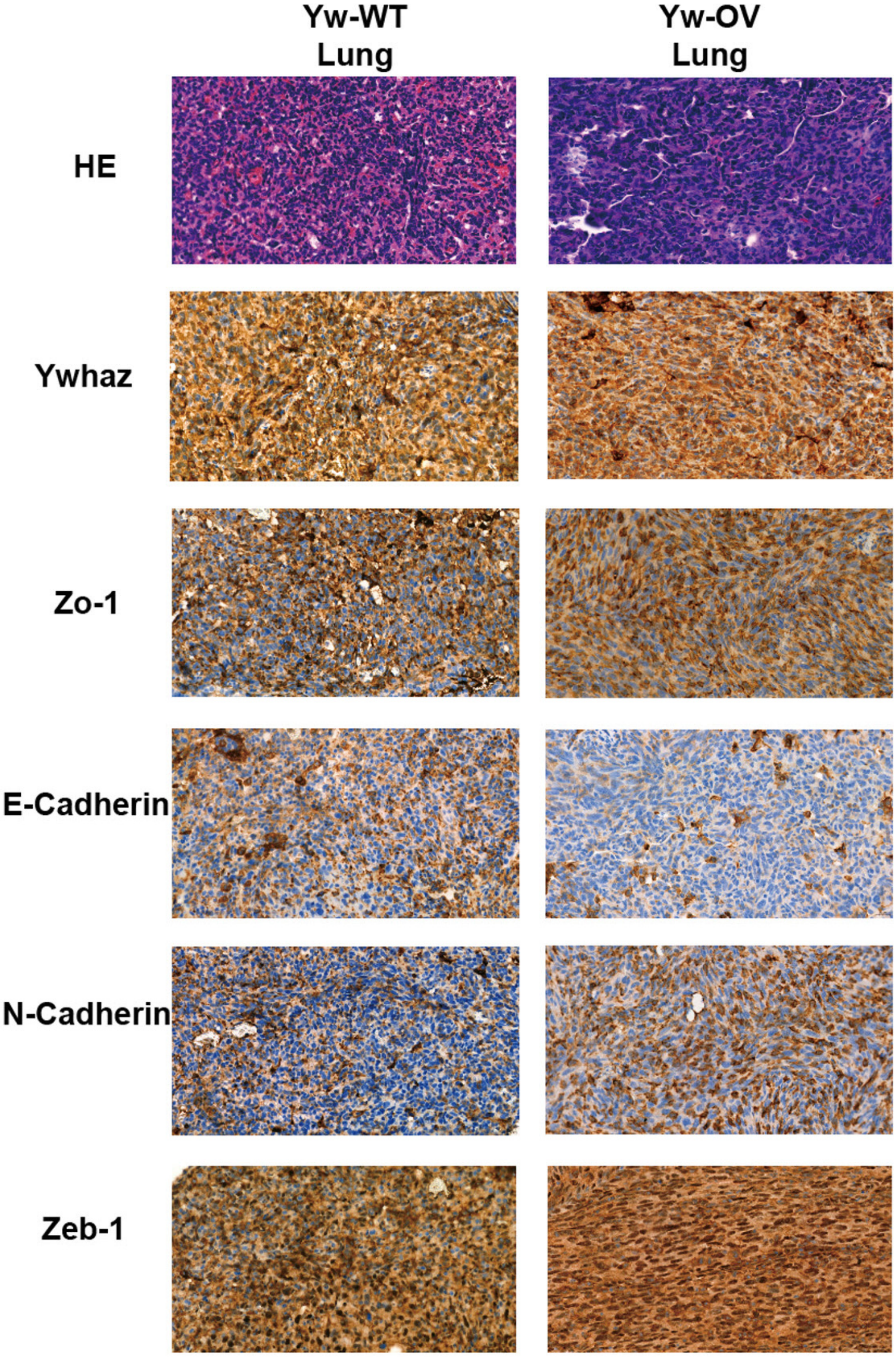

Supplementary Table S1. Primers used in this study

Name	Sequences	Application
ANXA3-3	AGCCACCAAAACTACTTAAAACCTCCAG	Splinkerette PCR
ANXA3-5	TAAACTAACCACATGCTGGCAACCTC	Splinkerette PCR
ERRFI1-3	TGAAACTGAGTCTTTATTGCCACTTCTCC	Splinkerette PCR
ERRFI1-5	TCACCCACTCCTCCTTTCTATCGTCA	Splinkerette PCR
YWHAZ-3	CTGTTCTGGACACTGCTCATTTGGCTAC	Splinkerette PCR
YWHAZ-5	TACTTGAGACGACCCTCCACGATGAC	Splinkerette PCR
ANXA2-3	GGATGTCTTTGAACTTCTGATCCTCCTG	Splinkerette PCR
ANXA2-5	GCATCGCAGAACTATGTCCAACTCCA	Splinkerette PCR
VMP1-3	GCACGTTGGCTTGACCTGGCTTACAC	Splinkerette PCR
VMP1-5	ACAGTTCAGTGTTGGTTGCAGGTGTT	Splinkerette PCR
HMSpAa	CGAAGAGTAACCGTTGCTAGGAGAGACCGTGGCTGAATGAGACT GGTGTCGACACTAGTGG	Splinkerette PCR
HMSpBb-Sau3A1	GATCCCACTAGTGTCGACACCAGTCTCTAATTTTTTTTTTCAAAAAAA	Splinkerette PCR
HMSp1	CGAAGAGTAACCGTTGCTAGGAGAGACC	Splinkerette PCR
HMSp2	GTGGCTGAATGAGACTGGTGTCGAC	Splinkerette PCR
PB3'-1st round	TAAATAAACCTCGATATACAGACCGATAAA	Splinkerette PCR
PB3'-2nd round	ATATACAGACCGATAAAACACATGCGTCAA	Splinkerette PCR
PB3'-seq	TTTTACGCATGATTATCTTTAACGTACGTC	Splinkerette PCR
PB5'-1st round	CAAAATCAGTGACACTTACCGCATTGACAA	Splinkerette PCR
PB5'-2nd round	CTTACCGCATTGACAAGCACGCCTCACGGG	Splinkerette PCR
PB5'-seq	TTAGAAAGAGAGAGCAATATTTCAAGAATG	Splinkerette PCR
E-cadherin-S	AGGTTTTCGGGCACCACTTA	qPCR
E-cadherin-AS	TGATGTTGCTGTCCCCAAGT	qPCR
CK19-S	TCCCAGCTCAGCATGAAAGCT	qPCR
CK19-AS	AAAACCGCTGATCACGCTCTG	qPCR
Zeb1-S	CCACTGTGGAGGACCAGAAT	qPCR
Zeb1-AS	CTCGTGAGGCCTCTTACCTG	qPCR
Fsp1-S	TTGTGTCCACCTTCCACA	qPCR
Fsp1-AS	GCTGTCCAAGTTGCTCAT	qPCR
N-cadherin-S	CATCAACCGGCTTAATGGTG	qPCR
N-cadherin-AS	ACTTTCACACGCAGGATGGA	qPCR
YWHAZ-S	GAAAAGTTCTTGATCCCCAATGC	qPCR
YWHAZ-AS	TGTGACTGGTCCACAATTCCTT	qPCR
GAPDH-S	ATGTTCCAGTATGACTCCACTCACG	qPCR
GAPDH-AS	GAAGACACCAGTAGACTCCACGACA	qPCR

Supplementary Table S2. The mouse models in the screening were summarized

Libraries	Mutant Clones	Injection methods	Mouse number per methods
L1	64,220	i.p.,i.v.,s.c.,o.r.	6
L2	39,666	i.p.,i.v.,s.c. o.r.	4
L3	64,357	i.p.,i.v.,s.c.,o.r.	6
L4	85,367	i.p.,i.v.,s.c.,o.r.	8*
L5	69,744	i.p.,i.v.,s.c.,o.r.	6
L6	94,743	i.p.,i.v.,s.c.,o.r.	9*
L7	43,690	i.p.,i.v.,s.c.,o.r.	4

"*" means two mice were missing in the orthotopic mouse models. The amount of cells injected was 2.5×10^6/mice for i.p., 1×10^6/mice for i.v., 2.5×10^6/mice for s.c. (both dorsal flanks), and 1×10^6/mice for o.r., respectively.

Supplementary Table S3. Metastatic subpopulation cultured from mice burdened with libraries

	Lung	Liver	Stomach	Adrenal	Brain	Metastasis in Cavity	Kidney	Gall Bladder	Total
i.p.	18	15	3	5	2	1	0	0	44
i.v.	19	1	0	0	2	9	0	0	31
o.r.	25	19	2	5	1	0	1	1	54
s.c.	20	2	0	1	4	3	0	0	30
Total									159

Supplementary Table S4. Highly metastatic tumor cells were dissected and cultured from both -/+ Dox treatment groups

	i.p.		o.r.		i.v.		s.c.		Total
Dox treatment	-	+	-	+	-	+	-	+	
Lung	5	6	5	6	16	9	9	9	65
Liver	4	3	3	4	0	1	1	0	16
Adrenal	2	2	1	2	0	0	0	0	7
Brain	1	1	1	2	1	1	2	0	9
Others*	0	0	0	0	4	2	3	1	10
Total	12	12	10	14	21	13	15	10	107

* means metastatic tumor in cavity and bone

Supplementary Table S5. Integration site analysis of the gene search vectors

PB-GSV(3TR) integration sites	Gene	Mutagenesis	PB-GSV Location in Gene	Organ Metastasis	Frequency	"-DOX(n,%)"	Mouse Model
chr4: 150,857,587	*Errfi1*	knock down	1st intron	lung, kidney, brain,bone, skin	26	19 (73.1%)	i.v.
chr11: 86,589,741	*Vmp1*	knock down	1st intron	lung, kidney, brain,bone, skin	26	19 (73.1%)	i.v.
chr4:150,857,587	*1700045H11Rik*	overexpress	downstream 1kb	lung, kidney, brain,bone, skin	26	19 (73.1%)	i.v.
chr9: 69,467,531	*Anxa2*	knock down	3rd intron	liver, kidney, adrenal,lung, brain	21	7 (33.3%)	o.r.
chr15: 36,791,535	*Ywhaz*	overexpress	2nd intron	lung, liver, kidney,brian, bone	20	15 (75%)	s.c.
chr5: 96,801,682	*Anxa3*	knock down	2nd intron	lung, liver	8	5 (62.5%)	i.p.
chr8: 123,433,321	*Def8*	overexpress	upstream 20kb	liver,lung,kidney	6	2(33.3%)	o.r.
chr9:16,296,965	*Fat3*	knock down	the last intron	lung	2	1(50%)	i.p./i.v.
chr16: 23,521,031	*Masp1*	overexpress	the promoter	peritoneal metastasis	1	1(100%)	s.c.
chr4: 45,912,953	*E230008N13Rik*	knock down	12th intron	lung	1	1(100%)	s.c.
chr2: 18,083,206	*Mllt10*	knock down	18th intron	lung	1	1(100%)	s.c.
chr9:123,431,934	*Lars2*	overexpress	10th intron	lung	1	1(100%)	i.v.
chr10: 13,746,456	*Aig1*	knock down	2nd intron	lung	1	1(100%)	i.v.
chr5: 142,906,409	*Actb*	overexpress	the last intron	lung	1	1(100%)	i.v.
chr3: 135,212,206	*Cenpe*	overexpress	in promoter	kidney	1	1(100%)	i.v.
chr15: 84,146,745	*Parvb*	knock down	downstream 1kb	lung	1	1(100%)	i.p.
chr6: 94,646,619	*Lrig1*	knock down	3rd intron	lung	1	1(100%)	i.p.
chr6: 94,646,619	*Slc25a26*	knock down	downstream 30kb	lung	1	1(100%)	i.p.
chr11: 78,264,792	*2610507B11Rik*	overexpress	5th intron	kidney	1	1(100%)	o.r.
chr15: 103,830,902	*Mucl1*	knock down	2nd intron	lung	1	1(100%)	o.r.
chr14: 63,537,545	*Mtmr9*	overexpress	the 5th intron	peritoneal metastasis	1	0(0)	i.v.
chr5: 110,058,064	*Gtpbp6*	knock down	upstream 30bp	peritoneal metastasis	1	0(0)	i.v.
chr5: 110,058,064	*Plcxd1*	overexpress	upstream 30kb	peritoneal metastasis	1	0(0)	i.v.
chr4:83,450,782	*Snapc3*	knock down	7th intron	peritoneal metastasis	1	0(0)	i.v.
chrX: 71,215,884	*Mtm1*	overexpress	2nd intron	lung	1	0(0)	i.v.
chr6: 70,782,214	*Rpia*	knock down	6th intron	peritoneal metastasis	1	0(0)	i.v.
chr3: 101,169,815	*Ptgfrn*	knock down	downstream 30kb	lung	1	0(0)	i.v.
chr16: 44,452,992	*Wdr52*	overexpress	17th intron	lung	1	0(0)	i.v.
chr8: 47,796,856	*Cdkn2aip*	knock down	downstream 30kb	lung	1	0(0)	i.v.
chr9: 118,359,311	*Eomes*	overexpress	upstream 30kb	lung	1	0(0)	i.v.
chr9: 118,359,311	*4933432G23Rik*	overexpress	upstream 30kb	lung	1	0(0)	i.v.
chr1: 156,605,921	*Abl2*	overexpress	2nd intron	lung	1	0(0)	i.v.
chr1: 150,099,386	*Ptgs2os*	overexpress	in promoter	lung	1	0(0)	i.v.
chr6: 83,465,612	*Dguok*	overexpress	the 5th intron	peritoneal metastasis	1	0(0)	i.v.
chr3: 87,150,664	*Kirrel*	overexpress	the last intron	lung	1	0(0)	s.c.
chr4: 149,779,407	*Slc25a33*	knock down	downstream 5kb	lung	1	0(0)	s.c.
chr16: 11,202,249	*2610020C07Rik*	overexpress	upstream 500bp	kidney	1	0(0)	o.r.
chr16: 11,202,249	*Rsl1d1*	knock down	8th intron	kidney	1	0(0)	o.r.
chr8: 64,947,008	*Tmem192*	overexpress	in promoter	liver	1	0(0)	o.r.
chr1: 26,559,603	*4931408C20Rik*	knock down	downsrteam 30kb	liver	1	0(0)	o.r.
chr2: 50,444,298	positive strand	None known genes or RNA sequence identified in these genome areas(NGR)		lung	1	1(100%)	i.p.
chr6: 76,628,998	negative strand			lung	1	0(0)	o.r.
Chr14: 45,801,718	negative strand			brain	1	0(0)	o.r.
chr7: 62,611,638	positive strand			brain	1	0(0)	o.r.
chr2: 57,742,460	positive strand			kidney	1	0(0)	i.v.
chrUn_random:4,604,836	positive strand			liver	1	0(0)	s.c.

Supplementary Table S6. Candidate genes involved in 12 main functional pathways

**Signaling pathways involved by identified genes**	**Mainly from -Dox group**	**Mainly from +Dox group**
Cell-cell adhesion /integrin signaling	*Ywhaz, Vmp1actb Parvb, Fat3*	*Anxa2, Fat3*
Cell mobility/invasion regulation	*Actb, Parvb*	*Anxa2, Abl2*
Wnt/TGF-β pathway signaling	*Ywhaz, Mllt10*	
Receptor/ protein tyrosine kinase (RTK) signaling	*Errfi1, Lrig1, Mucl1*	*Abl2*
Cell cycle control	*Ywhaz,Cenpe*	*Cdkn2aip*
Apoptosis/autophagy signaling	*Ywhaz, Anxa3, Aig1, Lrig1 Vmp1*	*Tmem192 Abl2 Cdkn2aip*
Immune response regulation	*Anxa3, Masp1, Mucl1*	
Early embryogenesis and development	*Fat3*	*Kirrel, Abl2 Fat3, Eomes*
Stem cell maintenance pathway signaling	*Lrig1*	*Eomes*
Metabolic/mitochondrial related pathways	*Slc25a26, Dguok, Lars2*	*Slc25a33, Rpia*
PTEN/phosphoinositide 3-kinase signaling	*Errfi1*	*Mtm1 and Mtmr9*
Gene expression and protein synthesis		*Snapc3*
Genes/non-coding RNAs of unknown function in cancer metastasis	*2610507B11Rik*	*Mgtpbp6, Plcxd1 2610020C07Rik, 4931408C20Rik, Def-8, Ptgs2os, Wdr52, 4933432G23Rik, Ptgfrn, Rsl1d1*

Supplementary Table S7. Multivariate COX regression analysis of the candidate genes in patients

N=173, Pancreatic Adenocarcinoma, Compared with Overall Survival Time

Candidate genes	HR (95% CI)	*P* value
ERRFI1	1.51 (0.99 − 2.3)	0.054
VMP1	0.67 (0.43-1.06)	0.084
ANX A2	2.5 (1.65 − 3.79)	8.4e−06
YWHAZ	2.65 (1.68 − 4.16)	1.2e−05
ANX A3	2.57 (1.54 − 4.28)	0.00017
FAT3	0.79 (0.52 − 1.21)	0.28
MASP1	0.82 (0.53 − 1.26)	0.36
MLLT10	0.65 (0.43 − 1.01)	0.051
LARS2	0.87 (0.56 − 1.36)	0.53
AIG1	0.78 (0.49 − 1.22)	0.28
ACTB	1.59 (0.97 − 2.59)	0.063
CENPE	2.29 (1.49 − 3.54)	0.00011
PARVB	0.58 (0.39 − 0.89)	0.01
LRIG1	0.79 (0.52 − 1.18)	0.25
SLC25A26	0.7 (0.44 − 1.1)	0.12
MUCL1	0.54 (0.32 − 0.89)	0.014

*Variables including expression level of the candidate genes, patients' age, gender and TMN stage. Data were extracted from TCGA and HPA databases.

Supplementary Table S8. The genetic alterations of candidate genes in patients

	Positive cases/Tested cases, Percent (%)		
**Candidate Genes**	**Point Mutation**	**Copy Number Variation**	**Gene expression**
YWHAZ	26/1258, 2.07%	AMP, 6.5%	Overexpressed 9/168, 13.69%
ANXA3	19/1258, 1.51%	HOMDEL,2.8% AMP,0.7%	Overexpressed 5/168, 2.98%
ANXA2	24/1258, 1.91%	HOMDEL,0.9% AMP,0.7%	-
CENPE	41/1258, 3.26%	HOMDEL,6.4%	Overexpressed 7/168, 4.17%
PARVB	108/1258, 8.59%	AMP,2.0% HOMDEL,1.1%	Overexpressed, 4/168, 2.38%
MUCL1	15/1258, 1.19%	AMP,0.7% HOMDEL,1.8%	-

Note "-" represents no data available. AMP: amplification; HOMDEL: Homologous deletion. Data were extracted from the COSMIC database (https://cancer.sanger.ac.uk/cosmic) and cBioPortal for cancer genomics (www.cbioportal.org).
