# Supplementary figures and images for: High-throughput functional screen identifies YWHAZ as a key regulator of pancreatic cancer metastasis
Source: Cell Death Dis. 2023 Jul 14;14(7):431. doi: 10.1038/s41419-023-05951-5 (PMC10349114; doi:10.1038/s41419-023-05951-5)

Western blot raw data

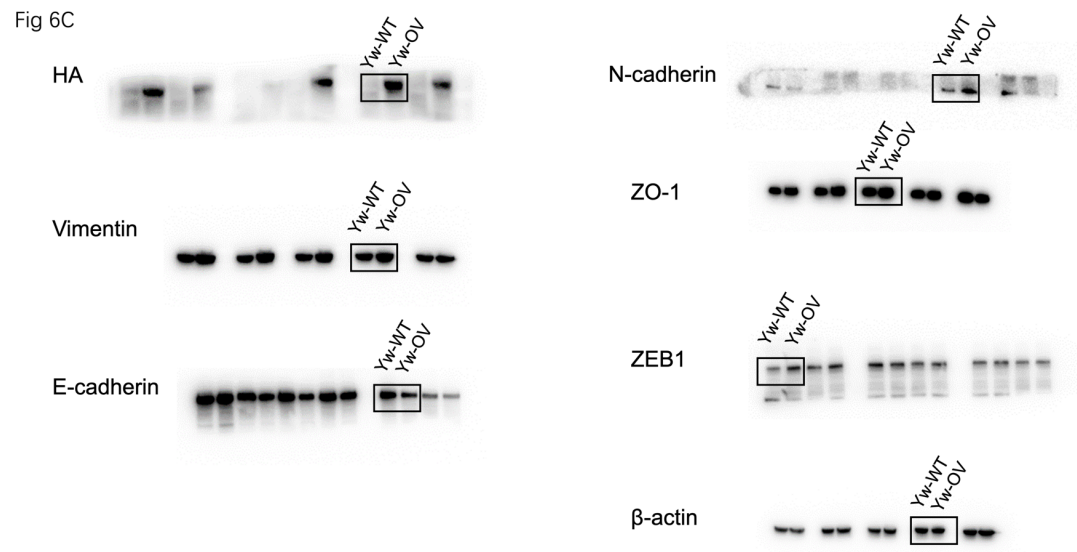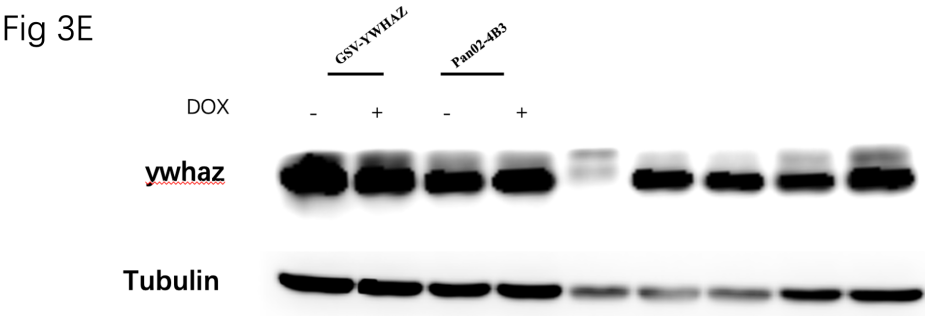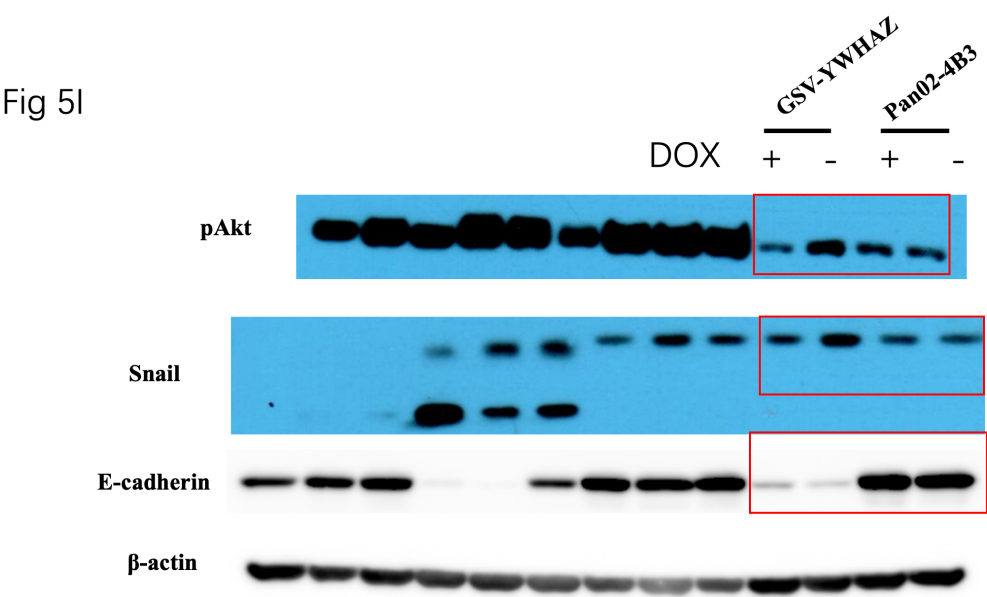

Supplement: Supplementary file 2 — Original Data File [file 41419_2023_5951_MOESM2_ESM.pdf]
