# Supplementary material for: High-throughput functional screen identifies YWHAZ as a key regulator of pancreatic cancer metastasis
Source: Cell Death Dis. 2023 Jul 14;14(7):431. doi: 10.1038/s41419-023-05951-5 (PMC10349114; doi:10.1038/s41419-023-05951-5)
Supplement: Supplementary file 3 — Response to the author list changes [file 41419_2023_5951_MOESM3_ESM.pdf]

YWHAZ story: Accepted in Principle

发起会议
2023-07-05 08:44:48

发件人： "kailongli" <kailongli@pku.edu.cn>

收件人： caofang820@163.com yunpengjiang@stu.pku.edu.cn changlin0730@126.com duhongzhen@ioz.ac.cn changde5501@foxmail.com chunxiaopan@outlook.com hxz182182@163.com yudonglin@stu.pku.edu.cn 497733742@qq.com ynfan2013@163.com bianxiaocui@ibms.cams.cn [↑隐藏信息]

发送成功 [查看详情] 共发给11个收件人，其中 11个成功到达对方服务器

Hi all,

Our manuscript entitled "High-throughput functional screen identifies YWHAZ as a key regulator of pancreatic cancer metastasis" has been provisionally accepted for publication in *Cell Death & Disease*. Our most recent author list differs from the one in the original submission because of the revision. To move forward, please **reply "Agree" to my email** confirming that you agree to these changes. Thanks.

The current list is:

Fang Cao[1,6], Yunpeng Jiang[5,6], Lin Chang[2,3], Hongzhen Du[3], De Chang[4], Chunxiao Pan[3], Xiaozheng Huang[1], Donglin Yu[5], Mi Zhang[4], Yongna Fan[3], Xiaocui Bian[3*], Kailong Li[5*]

Best,
Kailong

1.

Re: YWHAZ story: Accepted in Principle

发件人： "曹放" <caofang820@163.com>

收件人： "kailongli" <kailongli@pku.edu.cn>

Agree

---- Replied Message ----

2.

Re:YWHAZ story: Accepted in Principle

发件人： "蒋云鹏" <yunpengjiang@stu.pku.edu.cn>

收件人： "kailongli" <kailongli@pku.edu.cn>

Agree

3.

Re:YWHAZ story: Accepted in Principle

发件人： "linchang" <changlin0730@126.com>

收件人： "kailongli" <kailongli@pku.edu.cn>

抄 送： caofang820@163.com yunpengjiang@stu.pku.edu.cn

Agree

4.

Re: YWHAZ story: Accepted in Principle

发件人： "杜洪震" <duhongzhen@ioz.ac.cn>

收件人： "kailongli" <kailongli@pku.edu.cn>

Agree

5.

Re:YWHAZ story: Accepted in Principle

发件人： "Chandler" <changde5501@foxmail.com>

收件人： "kailongli" <kailongli@pku.edu.cn>

Agree.

6.

Re: YWHAZ story: Accepted in Principle

发件人： "chunxiaopan@outlook.com" <chunxiaopan@outlook.com>

收件人： "kailongli" <kailongli@pku.edu.cn> "caofang820" <caofang820@163.com>

.. [↓还有6个联系人]

Agree.

7.

Re: YWHAZ story: Accepted in Principle

发件人： "hxz182182" <hxz182182@163.com>

收件人： "kailongli" <kailongli@pku.edu.cn>

agree

8.

Re:YWHAZ story: Accepted in Principle

发件人： "余东林" <yudonglin@stu.pku.edu.cn>

收件人： "kailongli" <kailongli@pku.edu.cn>

抄 送： caofang820@163.com yunpengjiang@stu.pku.edu.cn

Agree

9.

Re: YWHAZ story: Accepted in Principle

发件人： "Mi Zhang" <497733742@qq.com>

收件人： "kailongli" <kailongli@pku.edu.cn>

Agree

10.

Re:YWHAZ story: Accepted in Principle

发件人： "范永娜" <ynfan2013@163.com>

收件人： "kailongli" <kailongli@pku.edu.cn>

Agree

11.

Re: YWHAZ story: Accepted in Principle

发件人： "卞晓翠" <bianxiaocui@ibms.cams.cn>

收件人： "kailongli" <kailongli@pku.edu.cn>

Agree
